# DNA metabarcoding provides insights into seasonal diet variations in Chinese mole shrew (*Anourosorex squamipes*) with potential implications for evaluating crop impacts

**DOI:** 10.1002/ece3.7055

**Published:** 2020-11-25

**Authors:** Ke‐yi Tang, Fei Xie, Hong‐yi Liu, Ying‐ting Pu, Dan Chen, Bo‐xin Qin, Chang‐kun Fu, Qiong Wang, Shun‐de Chen, Ke‐ji Guo

**Affiliations:** ^1^ College of Life Sciences Sichuan Normal University Chengdu China; ^2^ College of Biology and the Environment Nanjing Forestry University Nanjing China; ^3^ Central South Inventory and Planning Institute of National Forestry and Grassland Administration Changsha China

**Keywords:** Chinese mole shrew, ecology of pest, metabarcoding, molecular diet analysis, seasonal diet changes

## Abstract

Diet analysis of potential small mammals pest species is important for understanding feeding ecology and evaluating their impact on crops and stored foods. Chinese mole shrew (*Anourosorex squamipes*), distributed in Southwest China, has previously been reported as a farmland pest. Effective population management of this species requires a better understanding of its diet, which can be difficult to determine with high taxonomic resolution using conventional microhistological methods. In this study, we used two DNA metabarcoding assays to identify 38 animal species and 65 plant genera from shrew stomach contents, which suggest that *A. squamipes* is an omnivorous generalist. Earthworms are the most prevalent (>90%) and abundant (>80%) food items in the diverse diet of *A. squamipes*. Species of the Fabaceae (frequency of occurrence [FO]: 88%; such as peanuts) and Poaceae (FO: 71%; such as rice) families were the most common plant foods identified in the diet of *A. squamipes*. Additionally, we found a seasonal decrease in the diversity and abundance of invertebrate foods from spring and summer to winter. Chinese mole shrew has a diverse and flexible diet throughout the year to adapt to seasonal variations in food availability, contributing to its survival even when food resources are limited. This study provides a higher resolution identification of the diet of *A. squamipes* than has been previously described and is valuable for understanding shrew feeding ecology as well as evaluating possible species impacts on crops.

## Graphical abstract

Little is known about the invertebrate prey species and plants (especially crops at the species level) consumed by *A. squamipes*, which prevents understanding of their feeding ecology and thus impedes effective pest control. The Chinese mole shrew tends to be a generalized predator of a diverse array of invertebrates (e.g., earthworm) and plants (e.g., crops). Based on molecular techniques, diverse plant materials at the species level were identified with frequent observation of several important crops (e.g., rice and peanuts), indicating that *A. squamipes* could cause serious damage to crops or stored grains. Characterizing the diet of *A. squamipes* may have implications for the evaluating crop impacts and control of this shrew pest.

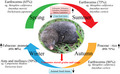

## 1 INTRODUCTION

The Chinese mole shrew (*Anourosorex squamipes* Milne‐Edwards, 1872) is a small insectivore mammal (He et al., 2016; Hoffmann, 1987; Motokawa et al., 2003), distributed in southwestern China and adjacent areas (He et al., 2016; Motokawa et al., 2003; Motokawa & Lin, 2002; Wilson et al., 2018). Like other house shrews (Khanam et al., 2016), *A. squamipes* is regarded as a pest in the agricultural ecosystem (Peng et al., 2018; Zong et al., 2017), causing both direct and indirect effects (Mdangi et al., 2013). As is widely known, synanthropic species association with human habitats widely impact agriculture and human health through damage to crops and transmission of pathogens (Khanam et al., 2016; Palis et al., 2007). The Chinese mole shrew affects crops and human health in China in a multitude of ways (Peng et al., 2018; Yang et al., 2013). For example, this shrew species consumes and contaminates stored grains and crops (Peng et al., 2018). In addition, it is considered to be a potential source of various types of viruses and pathogens (Gu et al., 2016; Song et al., 2007). *A. squamipes* caused severe damages to crops resulting from increased population sizes in Southwest China, especially in Sichuan Basin (Yang et al., 2013; Zong et al., 2017). Moreover, due to their peculiar food and foraging habits, existing rodent control practices are not suitable for controlling the number of these shrews, resulting in grain insecurity and reduced villager livelihoods.

Diet analysis are important for understanding how animal populations respond to resource distribution and variety as well as how to manage them (Gordon et al., 2019). Dietary information has been used in addition to pure feeding ecology in a variety of applied studies (Gong et al., 2017). Accurate identification of foods is a prerequisite to fully understanding the feeding ecology of a species and effectively controlling pest numbers (Heroldova et al., 2008). Better understanding of the feeding habits of house shrews can help to evaluate how growing populations of *A. squamipes* affect human and agricultural systems even during resource‐poor seasons and develop more effective pests management strategies, including targeted baits and lures (Khanam et al., 2016; Lathiya et al., 2008). However, very few studies have described the composition and seasonal variations in the Chinese mole shrew diet with higher taxonomic resolution.

For natural populations, it is difficult to accurately and efficiently assess wildlife diets, because of their elusive predatory behaviors and versatile feeding habits (Gong et al., 2017; Ozaki et al., 2018). Identifying food items with the highest taxonomic resolution is nearly impossible with traditional microhistological analysis of gut contents and stable isotope analysis (Jeunen et al., 2019; Murray et al., 2016; Rytkonen et al., 2019). A major limitation of the classical observational methods is that foods items are often digested to a greater extent, making identification of their remains taxonomically challenging (Berry et al., 2017; Bessey et al., 2019). Especially in the cases of insectivorous predators, whose prey is variable, small in size, and easily disintegrated in the gut, direct identification is difficult since their chyme contains a mixture of degraded prey fragments (Clare et al., 2014; Rytkonen et al., 2019). Besides, the stable isotope approach is unable to distinguish prey at the species level (Bohmann et al., 2018). Therefore, a broad‐spectrum technique with higher taxonomic resolution is necessary because shrew species have highly diverse and flexible diets that include insects, annelids, and plants (Churchfield et al., 2010, 2012; Haberl, 2002).

Here, DNA metabarcoding enabled us to identify food DNA (including highly degraded DNA) in gut contents or fecal samples with higher taxonomic resolution (Kartzinel & Pringle, 2015; Pompanon et al., 2012). To date, among shrew species, only the diets of Asian musk shrew (*Suncus murinus*) have been examined through DNA metabarcoding methods (Brown et al., 2014; Khanam et al., 2016). Most previous studies (Churchfield et al., 2010, 2012; De Pascual & De Ascencao, 2000; Haberl, 2002; McCay & Storm, 1997) that assessed diets in shrew species are based on microhistological identification of insect fragments in stomach contents or fecal pellets, resulting in large proportions of poorly resolved plant taxa and dietary information mainly at higher taxonomic levels. Little is known about the invertebrate prey species and plants (especially at the species level) consumed by Chinese mole shrew, which prevents understanding of their feeding ecology and thus impedes effective pest control.

In this study, we attempted to characterize the Chinese mole shrew diet across the four seasons by DNA metabarcoding of stomach samples. We compared dietary richness and composition across seasons to evaluate the impacts of this pest on crops and enhance our understanding of dietary breadth and seasonal food preferences in *A. squamipes*. Thus, this study may have implications for food niche and management of Chinese mole shrew as well as help to develop appropriate pest control strategies.

## 2 MATERIALS AND METHODS

### 2.1 Animal trapping

The animal samples of Chinese mole shrew were trapped from four seasons (Jan, Apr, Jul and Oct) from 2018 to 2019 in Pengzhou, Sichuan Province, southwest China. The sampling sites occupy a range of elevations from 515 to 575 m, longitude from 103.80°E to 104.10°E, and latitudes from 30.96°N to 31.12°N. All collected specimens were identified based on external characteristics in the field and were further confirmed according to skull morphology in the laboratory. As soon as animal specimens were collected, the animals were immediately stored at 0–4°C in the incubator with ice bags for transportation. The luminal stomach contents were collected at a super‐clean bench. And stomach contents were stored at −80°C for DNA extraction. Body mass is often used as a proxy for age in animals in previous study (Lavrinienko et al., 2018). Age identification method for *A. squamipes* referred to Yang et al. (2013). We followed the weight division criteria: Youth group (1) is less than 23.0 g, sub‐adult, group (2) was 23.1–28.0 g, adult group (3) was 28.1–38.0 g, and old group (4) was more than 38.0 g. A total of seventy‐two of adult Chinese mole shrew (18 in each season) were used in this study.

### 2.2 Stomach content samples and DNA extraction

The all samples of Chinese mole shrew were thawed at room temperature. We obtained stomach contents (SCs) collected from 72 individuals. We try to collect the foods in the interior of SCs to avoid the interference of host tissues or cells. The SCs samples were collected according to the guidelines and approval of the Animal Ethics Committee of Sichuan Normal University. After extracting from the stomach, the SCs was washed with ultrapure water and wiped off with other tissue between each extraction. Each SC was homogenized and stored in 95% ethanol for DNA extraction. The SCs samples of three individuals derived from the same field or woodland are homogeneously mixed into a mixed sample. Herein, six mixed SCs samples are used for further sequencing in each season, and a total of 24 mixed SCs samples are used for further molecular dietary analysis.

We extracted DNA used the QIAamp Fast DNA Stool Mini Kit (ID: 51604, QIAGEN), which is specifically developed for fecal and gut contents samples, according to the manufacturer's instructions. An extraction blank was included at each extraction series. The extracted DNA was further concentrated by evaporating samples in vacuum and then was stored for metabarcoding analysis.

### 2.3 Dietary DNA amplification and sequencing

PCR amplification was carried out using mitochondrial COΙ‐targeting primer (LCO‐1490/Uni‐MiniBar‐R), which produced a COΙ (cytochrome oxidase Ι) amplicon of 177 bp (Brown et al., 2014) for animal identification. Existing COΙ‐based approaches is widely preferred to identify unknown arthropod sequences (Zeale et al., 2011). The used primers were tested against Chinese mole shrew sequences to ensure no significant amplification of host DNA. And the rbcL (ribulose‐bisphosphate carboxylase gene) primers (h1aF and h2aR primers) were used to identify the plant species (Pierre et al., 2007). Sample specific barcode sequences were added to the COΙ and rbcL primers.

PCR were performed with PCR Using Q5® High‐Fidelity DNA Polymerase (M0491, NEB) according to the manufacturer's instruction. And PCR protocols were conducted primarily following Bohmann et al. (2018). Blank extraction controls were included on each PCR plate and for each different primer set. PCR products were then purified using a PCR purification kit (AXYGEN). Taking the purified PCR product as the template, quantitative real‐time PCR was performed on a Microplate reader (BioTek, FLx800) using Quant‐iT PicoGreen dsDNA Assay Kit. The amplicons for each sample were then mixed and purified according to the next high throughput sequencing requirements. Libraries for sequencing were constructed using the TruSeq Nano DNA LT Library Prep Kit (Illumina, San Diego, CA, United States) as recommended by the manufacturer. Libraries were sequenced on an Illumina Miseq platform (2 × 250 bp paired‐end reads) by Personalbio Bioinformatics Technology Corporation (Shanghai, China).

### 2.4 Sequence processing and data analysis

The raw reads were filtered through trimming and quality control steps prior to taxonomic assignment according to the QIIME v.1.7.0 quality control process (Caporaso et al., 2012). Adaptor/primer regions were removed, and potential chimeras were removed using USEARCHv9.2 (Edgar, 2013). Reads were clustered at 97% into Molecular Operational Taxonomic Units (MOTUs) according to the standard setting in USEARCHv9.2 (Edgar, 2013). Rarefaction curves were generated using QIIME v.1.7.0. and reached stable values, indicating that most of the species diversity were captured. High‐quality clean reads that passed quality filtering were queried against the full NCBI database using BLASTn according to previous study (Berry et al., 2017). MOTUs were resolved to species, genus, or higher, for COΙ animals or rbcL plants primer assays based on the percent similarity threshold: Sequences with identity ≥ 99% to a single species were considered as a “species match,” and as a “genus match” if sequences had ≥ 98% similarity to one or more species within the same genus. DNA sequences in this study were deposited into the NCBI Sequence Read Archive (SRA) under accession number: PRJNA637184.

Alpha diversity (i.e., Chao1, Shannon and Simpson) matrices were performed using QIIME and displayed using R v.3.3.3. software. To evaluate the pattern of dispersion of samples within each season, beta diversity was calculated with the euclidean distance. Beta diversity was calculated using QIIME and visualized by two‐dimensional principal coordinate analysis (PCoA). Diversity was compared between different seasons to assess temporal differences in diet composition. We also compared the relative abundance of food items at various taxonomic levels and at different seasons based on the linear discriminatory analysis (LDA) effect size (LEfSe) method using LEfSe software.

### 2.5 Statistical analyses

We used ANOVA to test for a significant difference in the dietary composition between different seasons. We also used a nonparametric statistical test (Kruskal–Wallis test) to assess the difference in alpha diversity index between different seasons. The frequency of occurrence (the number of pellets containing that foods divided by the total number of pellets in the species sample, FO) and the numbers of foods during different seasons were compared statistically using Dunnett's T3 multiple comparisons test by SPSS 20.0 software. The Mann–Whitney *U* test was also adopted to assess the difference in relative abundance of food items between different seasons following our previous study (Tang et al., 2019). Heat maps, box plots, and taxa summary bar charts were generated using the “ggplot2” package of R software (Wickham, 2009).

## 3 RESULTS

### 3.1 Overview of taxonomic assignment and dietary diversity

**Figure 1:**
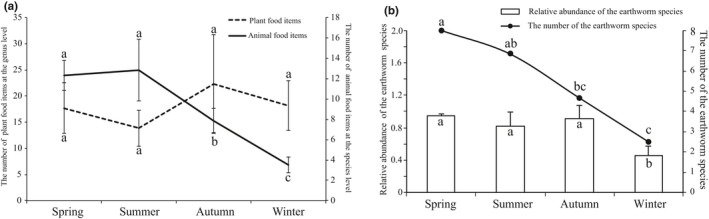
Seasonal variations in the Chinese mole shrew diet. (a) Seasonal changes in animal and plant food items at different taxonomic levels. (b) Seasonal changes in the numbers and relative abundances of earthworms at the species level. Different letters indicate a difference between seasons (*p* < .05)

In the 24 stomach samples analyzed over all seasons, the mean number of MOTUs in animal species was 38 ± 6 for spring, 30 ± 5 for summer, 32 ± 18 for autumn and 4 ± 1 for winter (Table 1). In plant food items, the mean number of MOTUs was 95 ± 28 for spring, 57 ± 24 for summer, 87 ± 41 for autumn, and 120 ± 42 for winter (Table 1). In total, we identified 38 potential animal food items (spanning 26 families and 15 orders) (Table S1) and 113 plant food items (spanning 39 families and 23 orders) (Table S2) at species level that are consumed by Chinese mole shrew. Seasonal dietary changes were detected in *A. squamipes* with a general shift toward low dietary diversity in winter. As expected, the number of animal food items at species level decreased significantly in winter (Figure 1a). Peak consumption of animal food items was detected in spring and summer, which were significantly higher than those in autumn and winter. However, we found no significant seasonal differences in the number of plant food items at genus level (Figure 1a), suggesting that potential plant food items were constant throughout the year.

**Table 1:** The number of Molecular Operational Taxonomic Units (MOTUs) and identified species of animal and plant food items in the Chinese mole shrew (*Anourosorex squamipes)* diet throughout the year

Food types	Identified level	Spring (Mean ± SE)	Summer (Mean ± SE)	Autumn (Mean ± SE)	Winter (Mean ± SE)
Animal (COI)	MOTUs	38 ± 6^a^	30 ± 5^a^	32 ± 18^ab^	4 ± 1^b^
	Assigned to species	12 ± 1^a^	13 ± 3^ab^	9 ± 1^b^	4 ± 1^c^
Plant (rbcL)	MOTUs	95 ± 28	57 ± 24	87 ± 41	120 ± 42
	Assigned to species	26 ± 7	17 ± 6	30 ± 13	30 ± 9

Note

Different letters indicate a difference between seasons (*p* < .05). A lack of superscript numbers denotes no significant difference. SE, standard error.

**Figure 2:**
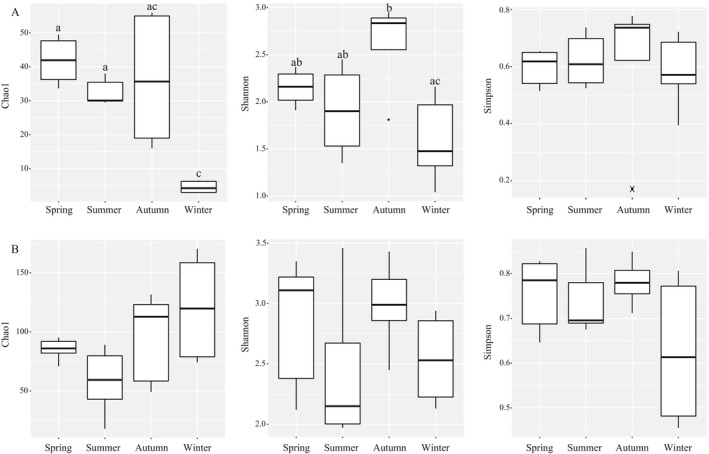
Box‐and‐whisker plots for alpha diversity in animal (a) and plant (b) food species estimators (Chao1, Shannon, and Simpson indices). Different letters indicate a difference between seasons (*p* < .05)

Alpha diversity indices (Chao1, Shannon, and Simpson) indicated seasonal differences in the diversity of animal food items. There was a significant greater Chao1 diversity index in spring and summer compared to winter (Figure 2a; *p* < .01). A higher Shannon diversity index was observed in autumn relative to winter (*p* < .05). No significant differences were found in Simpson index of animal food items (Figure 2a). Overall, our analysis showed a lower alpha diversity of animal food items in winter. However, the dietary alpha diversity of plant food items did not differ significantly between seasons (Figure 2b; *p* > .05), suggesting that the availability of plant‐derived foods were not affected by seasons.

**Figure 3:**
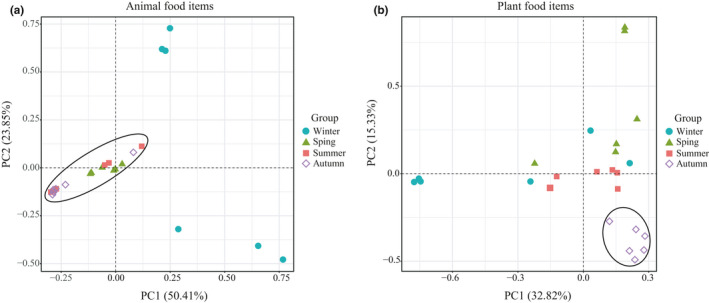
Two‐dimensional principal coordinate analysis (PCoA) of MOTUs of the Chinese mole shrew diet throughout the year. (a) represents animal food items and (b) represents plant food items. The first two principal coordinate (PC) axes are shown

The PCoA plot (Figure 3) revealed seasonal differences in animal‐derived diets. Animal food items in spring, summer, and autumn weakly clustered together and were separate from diets in winter (Figure 3a). In addition, there was dispersion within winter animal food items, suggesting a high degree of intragroup variability. We also observed a cluster of plant food items in autumn that was separated from those in other seasons with apparent dispersion (Figure 3b), suggesting a high degree of interindividual variability especially during winter. It could be explained by their opportunism and broad diet. The dominant family (Poaceae) in autumn likely contributed to this separation.

### 3.2 Dietary composition and seasonal variation in animal food items

We examined seasonal variations in the diet composition of *A. squamipes*, especially during times of resource limitation (e.g., in winter). Based on the full year, our results showed that although some small insects (ants, spiders, crickets, and beetles) were consumed, the Chinese mole shrew is primarily an earthworms‐eating shrew with a semi‐fossorial foraging mode. Using order‐level taxonomy only, species of Haplotaxida, Stylommatophora, Hymenoptera, Orthoptera, and Moniligastrida dominated the diet with species of Haplotaxida representing the highest FO (100%) and highest taxonomic richness (>74%) of consumption (Table S3). Notably, the consumption of Haplotaxida significant decreased (spring versus winter: 83% versus 45%, *p* = .002; summer versus winter: 81% versus 45%, *p* = .03; autumn versus winter: 90% versus 45%, *p* = .004) during winter (Table S3). Thus, earthworms were considered as the major food item in the diet of *A. squamipes*. In addition, as the common prey of shrews, arthropods (such as Orthoptera, Coleoptera, Dermaptera, Diptera, and Lepidoptera) were also detected but at low frequencies and relative abundances in *A. squamipes* diet (Table S3).

**Figure 4:**
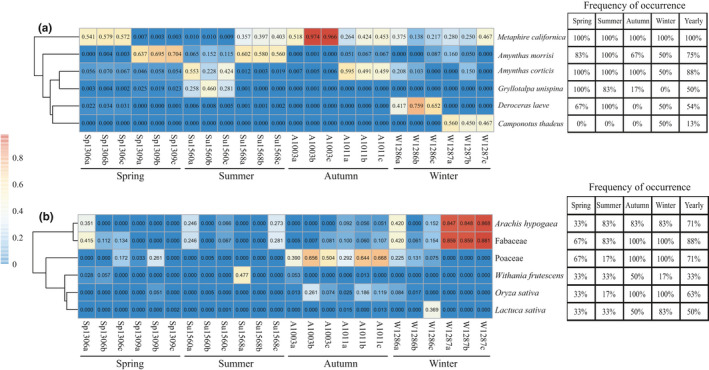
Heat map and FO of predominant animal (a) and plant (b) food items throughout the year. Each number in the heat map indicates the relative abundance of the corresponding food. Abbreviations: Sp, spring; Su, summer; A, autumn; W, winter

**Figure 5:**
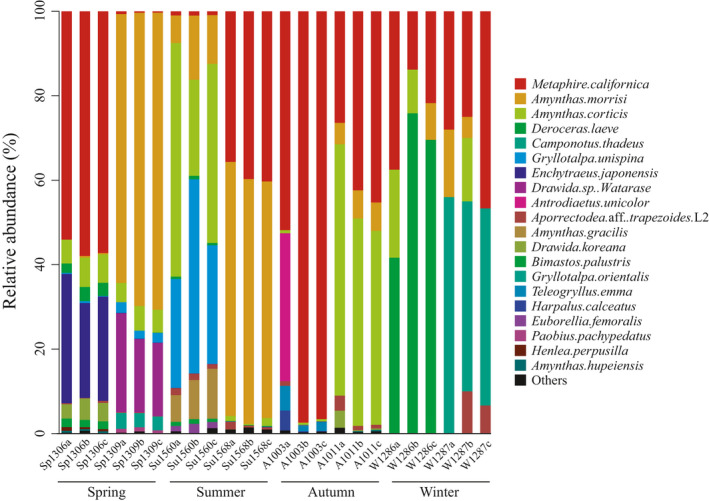
Relative abundance of the top 10 animal food items at the species level based on the COI metabarcoding assay

At the species level, the dominant (top five) animal species in terms of both FO and relative abundance were *Metaphire californica*, *Amynthas morrisi*, *Amynthas corticis*, *Deroceras laeve*, and *Camponotus thadeus* (Figures 4a, 5 and Table 2). Among the total animal food items, 12 different species of earthworms belonging to four families (Megascolecidae, Enchytraeidae, Moniligastridae, and Lumbricidae) accounted for 70%−80% of the animal‐derived diet (Table 2, Figure 5 and Table S4), indicating that these soil invertebrates are extremely abundant and diverse in the studied region. Among them, *Metaphire californica* was most frequently detected in all samples, contributing 19.8%−60% of the relative abundance of overall prey consumption and peaking at 60% in autumn (Table 2 and Figure 5). The second‐richest prey (*Amynthas morrisi*) were eaten more frequently and made up a larger proportion (>34%) of the available prey in spring and summer than in autumn and winter (<5%; Manne–Whitney *U* test: *p* = .008). In addition, we found a trend in the consumption of earthworms that shifted from higher numbers of earthworms during spring and summer to lower levels during autumn and the least in winter (Figure 1b and Table S4). Thus, the relative abundances of earthworms consumed by Chinese mole shrew during winter were significantly decreased (*p* < .01; Figure 1b). Meanwhile, the relative abundances of the all earthworm species significantly decreased (*p* < .01) during winter (Figure 1b), because their availability of was reduced. Our analysis indicated the animal‐derived diets of *A. squamipes* contain a high prevalence and diversity of earthworms. However, during winter, Chinese mole shrew predominantly preyed on *Camponotus thadeus* and *Deroceras leave* with a high FO (50%) and in higher proportions compared to other seasons (Table 2, Figures 4a and 5). Therefore, our study revealed that Chinese mole shrews have a broad diet comprising many different invertebrates of various sizes (dominantly earthworms) based on COΙ metabarcoding approaches.

**Table 2:** Frequency of occurrence (FO) and relative abundance of the top 20 animal food items in the Chinese mole shrew diet

Target Taxon		Spring		Summer		Autumn		Winter		Yearly
Family level	Species level	Relative abundance	FO (*N* = 6)	Relative abundance	FO (*N* = 6)	Relative Abundance	FO (*N* = 6)	Relative Abundance	FO (*N* = 6)	FO (*N* = 24)
Megascolecidae	*Metaphire californica*	0.284	100%	0.198	100%	0.600	100%	0.288	100%	100%
	*Amynthas morrisi*	0.340	83%	0.346	100%	0.031	67%	0.049	50%	75%
	*Amynthas corticis*	0.059	100%	0.206	100%	0.261	100%	0.077	50%	88%
	*Amynthas hupeiensis*	0.002	50%	0	0%	0.001	33%	0	0%	21%
	*Amynthas gracilis*	0.001	17%	0.046	67%	0.001	17%	0	0%	25%
	*Euborellia femoralis*	0	0%	0.008	50%	0	0%	0	0%	13%
Agriolimacidae	*Deroceras laeve*	0.015	67%	0.004	100%	0	0%	0.305	50%	54%
Formicidae	*Camponotus thadeus*	0	0%	0	0%	0	0%	0.246	50%	13%
Gryllotalpida	*Gryllotalpa unispina*	0.013	100%	0.167	83%	0.001	17%	0	0%	50%
Enchytraeidae	*Enchytraeus japonensis*	0.130	100%	0.001	67%	0.001	33%	0	0%	50%
	*Harpalus calceatus*	0	0%	0	0%	0.001	50%	0	0%	13%
Moniligastridae	*Drawida sp. Watarase*	0.097	67%	0.001	33%	0	0%	0	0%	25%
	*Drawida koreana*	0.022	67%	0.001	50%	0.008	17%	0	0%	33%
	*Ocnerodrilidae sp. 3 DP‐2015*	0	0%	0.002	50%	0	0%	0	0%	13%
Lumbricidae	*Bimastos palustris*	0.009	100%	0.005	50%	0	0%	0.007	17%	29%
	*Aporrectodea aff. Trapezoides*	0.002	100%	0.012	100%	0.011	67%	0.028	33%	75%
Antrodiaetidae	*Antrodiaetus unicolor*	0	0%	0	0%	0.058	17%	0	0%	4%
Anisolabididae	*Gryllotalpa orientalis*	0.017	100%	0	0%	0	0%	0	0%	13%
Carabidae	*Harpalus calceatus*	0	0%	0	0%	0.009	50%	0	0%	13%
Lithobiidae	*Teleogryllus emma*	0	0%	0	0%	0.015	50%	0	0%	13%

### 3.3 Dietary composition and seasonal variation in plant food items

**Figure 6:**
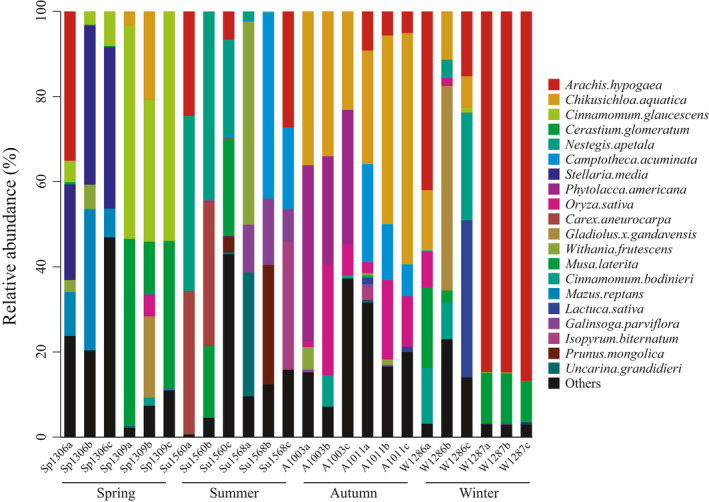
Relative abundance of the top 10 plant food items at the species level based on the rbcL metabarcoding assay

**Figure 7:**
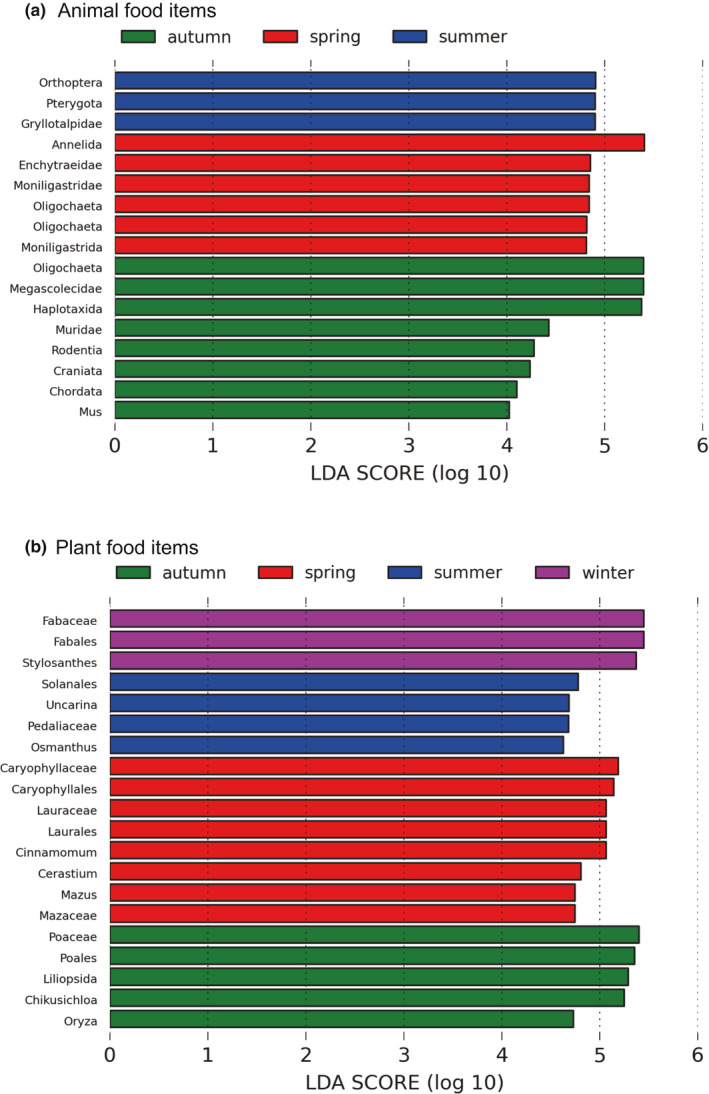
Animal (a) and plant (b) food items at taxonomic levels significantly differentiated between seasons as determined by linear discriminatory analysis (LDA) effect size (LEfSe). LDA scores were interpreted as the degree of difference in relative abundance

Generally, shrews are known to be small insectivorous mammals that preferentially target invertebrate prey. Interestingly, plant food items (especially crops) at various taxonomic levels were successfully detected from stomach contents of *A. squamipes* (Figures 6 and 7). The species of the Fabaceae family (FO: 88%) were the most common plant food items followed by Poaceae (FO: 71%) based on both FO and relative abundance (peak value > 50%) over the course of the year (Table 3). The winter plant‐derived diet of Chinese mole shrews was dominated by Fabaceae species (57.2% of plants consumed), with *Arachis hypogaea* (peanut) being the most frequently and abundantly eaten species from this family, representing 15.2%−86.8% of the identified plant food items (Figures 4b, 6 and Table S2). Poaceae species were found to significant increase in relative abundance during autumn (>53%) compared to other seasons (<8%) based on LEfSe analysis (Table 3 and Figure 7b), suggesting that *A. squamipes* feeds primarily on the seeds from Poaceae in autumn, peaking at 53% (Table 3 and Figure 4b). Oryza sativa (rice) as a commonly eaten crop species from the family Poaceae displayed the highest frequency (FO: 100%) and proportion (peaking at 26.1%), especially during postharvest period (e.g., autumn and winter; Figures 4b, 6 and Table S2). In addition, the crop species *Withania frutescens* (balsam pear) and *Lactuca sativa* (lettuce) were also identified during the year but contributed a very low percent of the plant diet (Figure 4b and Table S2). Our results confirmed that Chinese mole shrews could cause serious damage to crops or stored grains.

**Table 3:** The statistics of the top 10 plant taxa at the family level in the Chinese mole shrew diet throughout the year

Season	Spring			Summer			Autumn			Winter			Yearly	
Taxa	NO. of Occur. (*N* = 6)	FO (*N* = 6)	Relative abundance	NO. of Occur. (*N* = 6)	FO (*N* = 6)	Relative abundance	NO. of Occur. (*N* = 6)	FO (*N* = 6)	Relative abundance	NO. of Occur. (*N* = 6)	FO (*N* = 6)	Relative abundance	NO. of Occur. (*N* = 24)	FO (*N* = 24)
Fabaceae	4	67%	**0.111**	5	83%	**0.100**	6	100%	0.060	6	100%	**0.572**	21	88%
Poaceae	4	67%	0.080	1	17%	0.001	6	100%	**0.530**	6	100%	0.070	17	71%
Caryophyllaceae	6	100%	**0.315**	1	17%	<0.001	0	0%	0.000	5	83%	0.064	12	50%
Lauraceae	6	100%	**0.267**	3	50%	<0.001	5	83%	0.016	6	100%	0.074	20	83%
Oleaceae	2	33%	<0.001	4	67%	**0.198**	2	33%	<0.001	4	67%	0.006	12	50%
Asteraceae	3	50%	0.009	5	83%	**0.106**	4	67%	0.009	5	83%	0.053	17	71%
Nyssaceae	4	67%	<0.001	6	100%	**0.104**	5	83%	0.069	4	67%	0.001	19	79%
Phytolaccaceae	0	0%	0.000	0	0%	0.000	5	83%	**0.168**	1	17%	<0.001	6	25%
Iridaceae	1	17%	<0.001	0	0%	0.000	0	0%	0.000	1	17%	<0.001	2	8%
Solanaceae	2	33%	<0.001	2	33%	0.076	4	67%	<0.001	1	17%	0.039	9	38%
Cyperaceae	1	17%	0.016	3	50%	0.080	0	0%	0.000	1	17%	<0.001	5	21%

Bold values denote relative abundance of plant taxa >0.10.

Abbreviations: FO, Frequency of occurrence; No. of Occur., Number of occurrence.

Aside from crops, species from the Caryophyllaceae (31.5%) and Lauraceae (26.7%) families were also eaten by *A. squamipes* in higher proportions during spring compared to other seasons and appeared in all of the stomach contents samples (Table 3). *Chikusichloa aquatic*, which constituted the majority of Poaceae, was observed at a significant higher frequency (FO: 100%) and proportion (36.4%) in autumn compared to other seasons (Table S2). The Oleaceae, Asteraceae, and Nyssaceae were frequently observed during summer, accounting for 19.8%, 10.6%, and 10.4% of the identified plant diets, respectively (Table 3). In total, we observed high diversity in Chinese mole shrew plant‐derived diet throughout the year. A wide‐range foraging mode may explain the abundant numbers of this shrew even when food resources are limited during winter.

## 4 DISCUSSION

Our study utilized high‐resolution identification to explore the dietary compositions and seasonal diet variations of the Chinese mole shrew present in human habitats, aiming to increase understanding of shrew feeding ecology and evaluating their impact on the farming system. The Chinese mole shrew tends to be an opportunistic and generalized predator of a diverse array of invertebrates and plants, particularly earthworms and crops. With respect to common preys invertebrates, we confirm that Chinese mole shrew predominantly but not exclusively feeds on earthworms with a semi‐fossorial foraging mode similar to other shrews in temperate habitats (Churchfield et al., 2010, 2012; Khanam et al., 2016). Based on molecular technique, diverse plant materials at the species level were identified in the shrew stomach contents with frequent observation of several important crops (e.g., rice and peanut).

### 4.1 Characteristics of animal‐derived diet in the Chinese mole shrew

The diets of the Chinese mole shrew in our study are similar to the diets of other shrews (such as *Sorex* and *Blarina*) (Churchfield, 1982; Churchfield et al., 1999, 2012; Churchfield & Rychlik, 2006; Churchfield & Sheftel, 1994; De Pascual & De Ascencao, 2000), which include diverse invertebrates with a preponderance of earthworms (Table 2, Figures 1b, 5 and Table S1). The Chinese mole shrew can also be considered as an earthworm‐eating shrew. Using molecular technique, we obtained a sufficiently higher taxonomic resolution of food identification, especially earthworms (a total of 12 earthworm species were identified), compared to previous dietary analysis of shrews. Similar to early studies (Churchfield & Rychlik, 2006), many of the invertebrates eaten by *A. squamipes* are typical soil inhabitants (e.g., Oligochaeta and Formicidae), suggesting that this species of shrew is mainly subterranean in its foraging mode. Short‐tailed shrews are well adapted to a subterranean lifestyle and can push through soil and leaf litter with their long proboscis and elongated claws (Churchfield & Rychlik, 2006; Wu et al., 2011). These special morphological adaptations help to capture earthworms and ants depending on *A. squamipes* semi‐fossorial foraging behavior (He et al., 2016).

On the other hand, the preys of the Orthoptera, Formicidae, Coleoptera, Dermaptera, Diptera, and Lepidoptera families were occasionally observed during a particular season but only contributed a small amount of prey volume. Unlike the Chinese mole shrew, some other shrew species have been reported to predominantly feed on arthropods, not earthworms. For instance, Diptera (files), Formicidae (ants) and Araneae (spiders) were the most prey species among Southern short‐tailed shrew (*Blarina carolinensis*; Sylvester et al., 2012). The diet of European water shrew (*Neomys fodiens bicolor*) is composed mainly of lumbricids, isopods and dipterans (Churchfield, 2009). Isopterans (termites) and formicids were found to be the most frequent food items in the diet of elephant shrews (*Elephantulus myurus*; Churchfield, 1987). Lepidoptera larvae are the most common prey for masked shrew (*Sorex cinereus*) (Bellocq & Smith, 2003; McCay & Storm, 1997), followed by Coleoptera (beetles) and Aranea (spiders). The variations in diet compositions between different shrew species also imply that each one chooses what types of prey to feed on, presumably in relation to their morphological adaptations or according to the availability of food resources (Bellocq & Smith, 2003; De Pascual & De Ascencao, 2000).

### 4.2 Seasonal variations in animal‐derived diets in Chinese mole shrew

We also observed decreasing trends in diversity, proportions and FO of invertebrate consumption from spring to winter (Figure 1 and Table 1). One plausible explanation is the fact that seasonality has a strong effect on the density, biomass, and reproductive activity of the earthworm population (Kumar & Sabhlok, 2018; Monroy et al., 2006). For instance, the maximum density and mating activity of earthworms were achieved in spring (Biradar et al., 2008; Monroy et al., 2006). Furthermore, freezing weather and harsh climate conditions in winter influence the abundance and activity of food resources that can make it challenging for organisms to obtain sufficient amounts. For example, the activity of invertebrates is highly temperature‐dependent, and insect flight activity declines dramatically as the ambient temperature drops (Churchfield et al., 2012; Hope et al., 2014). In addition, a previous study showed that although earthworms were present in the soil profile in winter, their numbers and activity were sharply reduced (Khanam et al., 2016). In the case of snow cover and frozen soils, earthworms become dehydrated and hibernate (Churchfield et al., 2012). Randolph (1973) and Rozen (1988) also found that earthworm biomass clearly decreases from summer to winter. Moreover, McCay and Storm (1997) found that earthworms and other arthropods were more abundant in irrigated plots during both spring and autumn, suggesting greater availability of certain foods. Thus, earthworms may not be sufficiently abundant and available especially in winter. These findings strongly supported our results with respect to decreases in the proportions and numbers of earthworms consumed by *A. squamipes* during winter (Figure 1b). With their large surface‐area‐to‐volume ratios, short fasting endurance, and high metabolic rates, nonhibernating shrews need adequate food intake for maintaining endothermy and meeting high‐energy requirements at low temperatures (Brown et al., 2014; Churchfield et al., 2010, 2012). The increased consumption of relatively unpalatable and unprofitable prey, such as *Deroceras laeve* and *Camponotus thadeus*, in winter (Table 2 and Table S1) suggests that shrews are less preferential in winter than in summer, which is consistent with previous findings (Churchfield et al., 2012). Thus, the Chinese mole shrew selectively shifts its dietary preference throughout the year to adapt to seasonal foods resource availability.

### 4.3 Crop impacts due to Chinese mole shrews

Both plant and animal foods were detected in our study, indicating that *A. squamipes* may be an omnivorous generalist. No significant differences were detected in the numbers and alpha diversity of plant food items between the seasons (Figures 1a and 2), indicating that the availability of plant‐derived foods were balanced throughout the year. This opportunistic forager supplemented its diet with plant material, especially grains, in time of food shortages during winter when invertebrate preys are scarce (Figure 1 and Table 1). The Chinese mole shrew opts to feed on cultivated crops or stored grains (such as peanuts and rice) more often during autumn and winter because of the lack of more preferred prey, especially in winter (Figures 4b and 6). The reason for the abundance and high FOs of peanuts and rice in the diet may very well be their continued availability during autumn and winter. In southwest China, peanuts and rice are harvested during autumn. They are the staple food grains and stored for usage throughout the year. In addition, balsam pear and lettuce have been detected in the diet, suggesting that the Chinese mole shrew may cause damage to common vegetables in rural communities. Plant materials were also detected in the diet of several shrew species, such as armored shrew (Churchfield et al., 2010), and Southern short‐tailed shrew (Sylvester et al., 2012), and Asian musk shrew (Brown et al., 2014). However, very few studies have reported that shrews can cause damage to and contamination in grains. In this study, the proportional increase in crops eaten in autumn and winter suggests that the Chinese mole shrew poses a threat to crop production and grain stores (Figure 4b), especially in rice‐based farming systems. They have a vast geographic range, occupying a wide range of elevations from 300 to 4,000 m and latitudes from 18°N to 35°N (He et al., 2016; Motakawa et al., 2003). The Chinese mole shrews are abundant especially in Southwest China (He et al., 2016; Motakawa et al., 2003; Song et al., 2007), and the number of them showed an increasing trend in the study area (Liao et al., 2005; Zong et al., 2017). As a result, there may be potential negative impacts on agricultural production and people's health due to consumption and contamination of crops (Oyafuso, 2015). Therefore, development of methods to control the shrew populations on farmlands is necessary, and dietary analysis of *A. squamipes* can contribute to devising suitable poison baits.

Over 100 plant species were identified in stomach content of *A. squamipes*. Some of these may have been secondarily ingested via consumption of many large earthworms as reported by Churchfield et al. (2010). For *A. squamipes*, this dietary diversity may be a compensatory strategy to meet its high‐energy requirements by exploiting a wider variety of plant food items. However, a previous study also demonstrated that plant material (seeds or foliage) constitutes a smaller proportion of the overall shrew diet (Churchfield et al., 2012) as a result of missing data from highly digested plant foods. Thus, further investigation of shrew diet with higher taxonomic resolution is required to better understand the food composition of the species and determine their actual impact.

In summary, we found that *A. squamipes* has a diverse diet comprising a range of invertebrates and plant material. The single most important prey item, whether in terms of FOs, dietary composition or volume contribution, was earthworms. We revealed that the diet of this shrew contains a much higher prevalence and diversity of earthworms than previously known. We also found that plant materials (such as rice and peanuts) were consumed more frequently during the harvest season, implying that the Chinese mole shrew is omnivorous and play a pest role, despite being taxonomically classified as an insectivore. Therefore, the Chinese mole shrew is capable of shifting its dietary preferences to adapt to seasonal fluctuations of food resources, particularly during winter when the diversity and abundance of invertebrates are lowest. Characterizing the diet of *A. squamipes* may have implications for the evaluating crop impacts and control of this shrew species.

## CONFLICT OF INTEREST

The authors declare that they have no conflict of interest.

## AUTHOR CONTRIBUTION

**Keyi Tang:** Conceptualization (lead); Data curation (lead); Formal analysis (lead); Investigation (lead); Methodology (lead); Resources (lead); Software (lead); Writing‐original draft (lead); Writing‐review & editing (equal). **Fei Xie:** Investigation (equal); Resources (equal). **Hongyi Liu:** Data curation (supporting); Formal analysis (equal); Software (equal); Writing‐review & editing (equal). **Ying‐ting Pu:** Data curation (equal); Formal analysis (supporting); Software (equal); Visualization (equal); Writing‐original draft (equal). **Dan Chen:** Investigation (equal); Methodology (equal); Resources (equal). **Boxin Qin:** Investigation (equal); Methodology (equal); Resources (equal); Validation (equal). **Changkun Fu:** Data curation (equal); Formal analysis (equal); Investigation (equal); Resources (equal). **Qiong Wang:** Data curation (equal); Investigation (equal); Resources (equal); Software (equal). **Shunde Chen:** Conceptualization (lead); Data curation (equal); Funding acquisition (lead); Project administration (lead); Supervision (lead); Validation (lead); Visualization (lead); Writing‐original draft (supporting); Writing‐review & editing (lead). **Ke‐ji Guo:** Data curation (equal); Formal analysis (supporting); Software (equal); Supervision (equal); Visualization (equal); Writing‐review & editing (equal).

## Supporting information

Table S1

Table S2

Table S3

Table S4

## Data Availability

DNA sequences in this study were deposited into the NCBI Sequence Read Archive (SRA) under accession number: PRJNA637184. (https://www.ncbi.nlm.nih.gov/).
