# Supplementary material for: DNA metabarcoding provides insights into seasonal diet variations in Chinese mole shrew (Anourosorex squamipes) with potential implications for evaluating crop impacts
Source: Ecol Evol. 2020 Nov 25;11(1):376–89. doi: 10.1002/ece3.7055 (PMC7790647; doi:10.1002/ece3.7055)
Supplement: Supplementary file 1 — Table S1 [file ECE3-11-376-s001.doc]

Supplementary Table 1. FO (frequency of occurrence) of animal food items at the species level in the Chinese mole shrew diet throughout the year.

| Species-Level | Spring | Summer | Autumn | Winter | Yearly |
| --- | --- | --- | --- | --- | --- |
| *Metaphire californica* | 100% | 100% | 100% | 100% | 100% |
| *Amynthas morrisi* | 83% | 100% | 67% | 50% | 75% |
| *Amynthas corticis* | 100% | 100% | 100% | 50% | 88% |
| *Deroceras laeve* | 67% | 100% | 0% | 50% | 54% |
| *Camponotus thadeus* | 0% | 0% | 0% | 50% | 13% |
| *Gryllotalpa unispina* | 100% | 83% | 17% | 0% | 50% |
| *Enchytraeus japonensis* | 100% | 67% | 33% | 0% | 50% |
| *Drawida* sp. *Watarase* | 67% | 33% | 0% | 0% | 25% |
| *Antrodiaetus unicolor* | 0% | 0% | 17% | 0% | 4% |
| *Aporrectodea aff. trapezoides* | 100% | 100% | 67% | 33% | 75% |
| *Amynthas gracilis* | 17% | 67% | 17% | 0% | 25% |
| *Drawida koreana* | 67% | 17% | 50% | 0% | 33% |
| *Bimastos palustris* | 100% | 50% | 0% | 17% | 29% |
| *Gryllotalpa orientalis* | 100% | 0% | 0% | 0% | 13% |
| *Teleogryllus emma* | 0% | 0% | 50% | 0% | 13% |
| *Harpalus calceatus* | 0% | 0% | 50% | 0% | 13% |
| *Euborellia femoralis* | 0% | 50% | 0% | 0% | 13% |
| *Paobius pachypedatus* | 50% | 0% | 0% | 0% | 13% |
| *Henlea perpusilla* | 50% | 0% | 0% | 0% | 13% |
| *Amynthas hupeiensis* | 50% | 0% | 33% | 0% | 21% |
| *Nola cicatricalis* | 0% | 0% | 50% | 0% | 13% |
| *Anisodactylus binotatus* | 0% | 33% | 0% | 0% | 8% |
| *Angarotipula tumidicornis* | 0% | 0% | 67% | 0% | 17% |
| Ocnerodrilidae sp. 3 DP-2015 | 0% | 50% | 0% | 0% | 13% |
| *Mus musculus* | 17% | 17% | 67% | 0% | 25% |
| Sigmella sp. 1 ZW-2016 | 0% | 33% | 0% | 0% | 8% |
| Drawida sp. Monobe | 33% | 0% | 0% | 0% | 8% |
| *Grandidierella chaohuensis* | 67% | 67% | 0% | 0% | 38% |
| Plecia sp. SS-2012 | 50% | 0% | 0% | 0% | 13% |
| Dolichopodidae sp. BOLD-2016 | 17% | 0% | 0% | 0% | 4% |
| Megascolecidae sp. 2011-03 | 0% | 50% | 0% | 0% | 13% |
| *Gomphocerippus rufus* | 0% | 17% | 0% | 0% | 4% |
| *Physella acuta* | 0% | 50% | 0% | 0% | 13% |
| *Yaginumia sia* | 0% | 17% | 0% | 0% | 4% |
| *Psammotettix confinis* | 0% | 17% | 0% | 0% | 4% |
| *Stenobothrus lineatus* | 0% | 17% | 0% | 0% | 4% |
| *Agrotis ipsilon* | 0% | 17% | 0% | 0% | 4% |
| *Nysius thymi* | 0% | 17% | 0% | 0% | 4% |
