# Supplementary material for: DNA metabarcoding provides insights into seasonal diet variations in Chinese mole shrew (Anourosorex squamipes) with potential implications for evaluating crop impacts
Source: Ecol Evol. 2020 Nov 25;11(1):376–89. doi: 10.1002/ece3.7055 (PMC7790647; doi:10.1002/ece3.7055)
Supplement: Supplementary file 2 — Table S2 [file ECE3-11-376-s002.doc]

Supplementary Table 2. Relative abundance of plant food items at the species level in all Chinese mole shrew diet samples.

Supplementary Table 2.1 Relative abundance of plant food items at the species level in all Chinese mole shrew diet samples during spring.

| Taxon | Relative abundance (Spring) | | | | | |
| --- | --- | --- | --- | --- | --- | --- |
|  | Sp1306a | Sp1306b | Sp1306c | Sp1309a | Sp1309b | Sp1309c |
| f__Fabaceae;g__Arachis;s__*Arachis hypogaea* | 0.3508 | 0.0001 | 0.0000 | 0.0000 | 0.0000 | 0.0000 |
| f__Poaceae;g__Chikusichloa;s__*Chikusichloa aquatica* | 0.0000 | 0.0000 | 0.0000 | 0.0330 | 0.2087 | 0.0002 |
| f__Lauraceae;g__Cinnamomum;s__*Cinnamomum glaucescens* | 0.0506 | 0.0310 | 0.0813 | 0.5011 | 0.3321 | 0.5388 |
| f__Caryophyllaceae;g__Cerastium;s__*Cerastium glomeratum* | 0.0049 | 0.0020 | 0.0024 | 0.4403 | 0.1235 | 0.3467 |
| f__Oleaceae;g__Nestegis;s__*Nestegis apetala* | 0.0001 | 0.0000 | 0.0000 | 0.0000 | 0.0000 | 0.0001 |
| f__unidentified;g__Camptotheca;s__*Camptotheca acuminata* | 0.0000 | 0.0001 | 0.0002 | 0.0004 | 0.0003 | 0.0005 |
| f__Caryophyllaceae;g__Stellaria;s__*Stellaria media* | 0.2245 | 0.3737 | 0.3791 | 0.0013 | 0.0003 | 0.0005 |
| f__Phytolaccaceae;g__Phytolacca;s__*Phytolacca americana* | 0.0000 | 0.0000 | 0.0000 | 0.0000 | 0.0000 | 0.0000 |
| f__Poaceae;g__Oryza;s__*Oryza sativa* | 0.0000 | 0.0000 | 0.0000 | 0.0002 | 0.0514 | 0.0000 |
| f__Cyperaceae;g__Carex;s__*Carex aneurocarpa* | 0.0002 | 0.0000 | 0.0000 | 0.0000 | 0.0000 | 0.0000 |
| f__Iridaceae;g__Gladiolus;s__*Gladiolus x gandavensis* | 0.0000 | 0.0000 | 0.0000 | 0.0000 | 0.1906 | 0.0000 |
| f__Solanaceae;g__Withania;s__*Withania frutescens* | 0.0281 | 0.0574 | 0.0000 | 0.0000 | 0.0000 | 0.0000 |
| f__Musaceae;g__Musa;s__*Musa laterita* | 0.0000 | 0.0000 | 0.0000 | 0.0000 | 0.0000 | 0.0000 |
| f__Lauraceae;g__Cinnamomum;s__*Cinnamomum bodinieri* | 0.0001 | 0.0000 | 0.0000 | 0.0010 | 0.0192 | 0.0021 |
| f__Mazaceae;g__Mazus;s__*Mazus reptans* | 0.1031 | 0.3320 | 0.0679 | 0.0000 | 0.0000 | 0.0000 |
| f__Asteraceae;g__Lactuca;s__*Lactuca sativa* | 0.0001 | 0.0000 | 0.0000 | 0.0000 | 0.0000 | 0.0016 |
| f__Asteraceae;g__Galinsoga;s__*Galinsoga parviflora* | 0.0001 | 0.0000 | 0.0000 | 0.0000 | 0.0000 | 0.0001 |
| f__Ranunculaceae;g__Isopyrum;s__*Isopyrum biternatum* | 0.0000 | 0.0000 | 0.0000 | 0.0000 | 0.0000 | 0.0000 |
| f__Rosaceae;g__Prunus;s__*Prunus mongolica* | 0.0000 | 0.0000 | 0.0000 | 0.0000 | 0.0000 | 0.0000 |
| f__Pedaliaceae;g__Uncarina;s__*Uncarina grandidieri* | 0.0000 | 0.0000 | 0.0000 | 0.0000 | 0.0000 | 0.0000 |
| f__Fabaceae;g__Cyamopsis;s__*Cyamopsis tetragonoloba* | 0.0602 | 0.1042 | 0.1191 | 0.0001 | 0.0000 | 0.0000 |
| f__Linderniaceae;g__Lindernia;s__*Lindernia antipoda* | 0.0000 | 0.0000 | 0.0000 | 0.0000 | 0.0000 | 0.0000 |
| f__Asteraceae;g__Leontopodium;s__*Leontopodium alpinum* | 0.0000 | 0.0000 | 0.0000 | 0.0000 | 0.0000 | 0.0000 |
| f__Poaceae;g__Eleusine;s__*Eleusine indica* | 0.0000 | 0.0000 | 0.0048 | 0.0001 | 0.0000 | 0.0000 |
| f__Polygonaceae;g__Bistorta;s__*Bistorta vivipara* | 0.0000 | 0.0000 | 0.0000 | 0.0000 | 0.0000 | 0.0000 |
| f__Juglandaceae;g__Carya;s__*Carya glabra* | 0.0546 | 0.0000 | 0.0000 | 0.0000 | 0.0206 | 0.0000 |
| f__Poaceae;g__Orinus;s__*Orinus kokonoricus* | 0.0000 | 0.0000 | 0.1583 | 0.0000 | 0.0000 | 0.0000 |
| f__Fabaceae;g__Medicago;s__*Medicago sativa* | 0.0000 | 0.0000 | 0.0000 | 0.0000 | 0.0000 | 0.0000 |
| f__Alismataceae;g__Sagittaria;s__*Sagittaria trifolia* | 0.0000 | 0.0001 | 0.1230 | 0.0000 | 0.0000 | 0.0000 |
| f__Amaryllidaceae;g__Allium;s__*Allium stellatum* | 0.0000 | 0.0000 | 0.0000 | 0.0000 | 0.0000 | 0.0000 |
| f__Euphorbiaceae;g__Euphorbia;s__*Euphorbia hypericifolia* | 0.0000 | 0.0000 | 0.0000 | 0.0000 | 0.0000 | 0.0000 |
| f__Amaryllidaceae;g__Allium;s__*Allium prattii* | 0.0000 | 0.0000 | 0.0000 | 0.0000 | 0.0000 | 0.0000 |
| f__Euphorbiaceae;g__Acalypha;s__*Acalypha australis* | 0.0000 | 0.0000 | 0.0000 | 0.0000 | 0.0000 | 0.0000 |
| f__Asteraceae;g__Faberia;s__*Faberia pinnatifida* | 0.0000 | 0.0000 | 0.0000 | 0.0000 | 0.0000 | 0.0460 |
| f__Pylaisiadelphaceae;g__Pylaisiadelpha;s__*Pylaisiadelpha tenuirostris* | 0.0808 | 0.0000 | 0.0000 | 0.0000 | 0.0000 | 0.0000 |
| f__Apiaceae;g__Ligusticum;s__*Ligusticum sinense* | 0.0000 | 0.0000 | 0.0000 | 0.0017 | 0.0000 | 0.0000 |
| f__Rubiaceae;g__Uncaria;s__*Uncaria tomentosa* | 0.0000 | 0.0001 | 0.0000 | 0.0000 | 0.0000 | 0.0000 |
| f__Hypnaceae;g__Callicladium;s__*Callicladium haldanianum* | 0.0001 | 0.0000 | 0.0000 | 0.0000 | 0.0000 | 0.0000 |
| f__Fabaceae;g__Medicago;s__*Medicago laciniata* | 0.0000 | 0.0000 | 0.0000 | 0.0000 | 0.0000 | 0.0000 |
| f__Rosaceae;g__Rosa;s__*Rosa chinensis var. spontanea* | 0.0000 | 0.0000 | 0.0000 | 0.0000 | 0.0000 | 0.0000 |
| f__Rubiaceae;g__Ophiorrhiza;s__Ophiorrhiza sp. SH-2010 | 0.0092 | 0.0331 | 0.0085 | 0.0000 | 0.0000 | 0.0000 |
| f__Oleaceae;g__Osmanthus;s__*Osmanthus americanus* | 0.0000 | 0.0000 | 0.0000 | 0.0000 | 0.0000 | 0.0000 |
| f__Polygonaceae;g__Rumex;s__*Rumex utahensis* | 0.0000 | 0.0000 | 0.0000 | 0.0000 | 0.0000 | 0.0507 |
| f__Musaceae;g__Musa;s__*Musa acuminata* | 0.0000 | 0.0000 | 0.0000 | 0.0000 | 0.0000 | 0.0000 |
| f__Moraceae;g__Ficus;s__*Ficus benghalensis* | 0.0000 | 0.0000 | 0.0000 | 0.0000 | 0.0000 | 0.0000 |
| f__Cucurbitaceae;g__Cucurbita;s__*Cucurbita pepo* | 0.0000 | 0.0000 | 0.0000 | 0.0000 | 0.0000 | 0.0000 |
| f__Convolvulaceae;g__Convolvulus;s__*Convolvulus arvensis* | 0.0000 | 0.0000 | 0.0000 | 0.0000 | 0.0000 | 0.0000 |
| f__Poaceae;g__Oryza;s__*Oryza rhizomatis* | 0.0000 | 0.0000 | 0.0000 | 0.0000 | 0.0000 | 0.0000 |
| f__Brassicaceae;g__Brassica;s__*Brassica oleracea var. botrytis* | 0.0001 | 0.0000 | 0.0000 | 0.0143 | 0.0000 | 0.0115 |
| f__Asteraceae;g__Helichrysum;s__*Helichrysum zeyheri* | 0.0000 | 0.0000 | 0.0000 | 0.0000 | 0.0000 | 0.0002 |
| f__Araceae;g__Lemna;s__*Lemna minor* | 0.0000 | 0.0000 | 0.0000 | 0.0000 | 0.0000 | 0.0000 |
| f__Cornaceae;g__Cornus;s__*Cornus sericea* | 0.0152 | 0.0190 | 0.0060 | 0.0000 | 0.0000 | 0.0000 |
| f__Betulaceae;g__Alnus;s__*Alnus rubra* | 0.0000 | 0.0000 | 0.0000 | 0.0000 | 0.0348 | 0.0000 |
| f__Solanaceae;g__Capsicum;s__*Capsicum annuum* | 0.0030 | 0.0037 | 0.0000 | 0.0000 | 0.0000 | 0.0000 |
| f__Iridaceae;g__Gladiolus;s__*Gladiolus palustris* | 0.0000 | 0.0000 | 0.0000 | 0.0000 | 0.0157 | 0.0000 |
| f__Cucurbitaceae;g__Cucumis;s__*Cucumis sativus* | 0.0000 | 0.0000 | 0.0000 | 0.0000 | 0.0000 | 0.0000 |
| f__Cucurbitaceae;g__Cucurbita;s__*Cucurbita moschata* | 0.0000 | 0.0000 | 0.0000 | 0.0000 | 0.0000 | 0.0000 |
| f__Fabaceae;g__Glycine;s__*Glycine canescens* | 0.0014 | 0.0061 | 0.0132 | 0.0000 | 0.0000 | 0.0000 |
| f__Oxalidaceae;g__Oxalis;s__*Oxalis debilis* | 0.0000 | 0.0186 | 0.0000 | 0.0000 | 0.0000 | 0.0000 |
| f__Fabaceae;g__Kummerowia;s__*Kummerowia striata* | 0.0006 | 0.0000 | 0.0001 | 0.0000 | 0.0000 | 0.0000 |
| f__Fabaceae;g__Stylosanthes;s__*Stylosanthes viscosa* | 0.0003 | 0.0000 | 0.0000 | 0.0000 | 0.0000 | 0.0000 |
| f__Achatocarpaceae;g__Achatocarpus;s__*Achatocarpus gracilis* | 0.0000 | 0.0000 | 0.0112 | 0.0017 | 0.0013 | 0.0006 |
| f__Poaceae;g__Oryza;s__*Oryza longiglumis* | 0.0000 | 0.0000 | 0.0000 | 0.0000 | 0.0000 | 0.0000 |
| f__Fabaceae;g__Pterocarpus;s__*Pterocarpus ternatus* | 0.0010 | 0.0000 | 0.0000 | 0.0000 | 0.0000 | 0.0000 |
| f__Solanaceae;g__Lycium;s__*Lycium chinense* | 0.0032 | 0.0053 | 0.0000 | 0.0000 | 0.0000 | 0.0000 |
| f__Ranunculaceae;g__Isopyrum;s__*Isopyrum savilei* | 0.0000 | 0.0000 | 0.0000 | 0.0000 | 0.0000 | 0.0000 |
| f__Apiaceae;g__Chaerophyllum;s__*Chaerophyllum procumbens* | 0.0000 | 0.0000 | 0.0000 | 0.0000 | 0.0000 | 0.0000 |
| f__Brassicaceae;g__Draba;s__*Draba densifolia* | 0.0000 | 0.0000 | 0.0000 | 0.0001 | 0.0000 | 0.0000 |
| f__Caryophyllaceae;g__Cerastium;s__*Cerastium arvense* | 0.0005 | 0.0001 | 0.0001 | 0.0020 | 0.0000 | 0.0000 |
| f__Fabaceae;g__Craspedolobium;s__*Craspedolobium unijugum* | 0.0011 | 0.0015 | 0.0020 | 0.0000 | 0.0000 | 0.0000 |
| f__Caryophyllaceae;g__Stellaria;s__*Stellaria holostea* | 0.0006 | 0.0000 | 0.0000 | 0.0000 | 0.0000 | 0.0000 |
| f__Solanaceae;g__Solanum;s__*Solanum sp. Espinoza5665* | 0.0027 | 0.0016 | 0.0000 | 0.0000 | 0.0000 | 0.0000 |
| f__Rosaceae;g__Prunus;s__*Prunus takesimensis* | 0.0001 | 0.0000 | 0.0000 | 0.0000 | 0.0000 | 0.0000 |
| f__Caryophyllaceae;g__Stellaria;s__*Stellaria americana* | 0.0000 | 0.0001 | 0.0053 | 0.0000 | 0.0000 | 0.0000 |
| f__Poaceae;g__Ctenium;s__*Ctenium floridanum* | 0.0000 | 0.0000 | 0.0044 | 0.0000 | 0.0000 | 0.0000 |
| f__Oxalidaceae;g__Oxalis;s__*Oxalis violacea* | 0.0000 | 0.0051 | 0.0000 | 0.0000 | 0.0000 | 0.0000 |
| f__Amaranthaceae;g__Achyranthes;s__*Achyranthes aspera* | 0.0000 | 0.0000 | 0.0041 | 0.0000 | 0.0000 | 0.0000 |
| f__Cucurbitaceae;g__Luffa;s__*Luffa quinquefida* | 0.0000 | 0.0000 | 0.0000 | 0.0000 | 0.0000 | 0.0000 |
| f__Rutaceae;g__Zanthoxylum;s__*Zanthoxylum clava-herculis* | 0.0000 | 0.0000 | 0.0000 | 0.0000 | 0.0000 | 0.0000 |
| f__Polygonaceae;g__Bistorta;s__*Bistorta officinalis* | 0.0000 | 0.0000 | 0.0000 | 0.0000 | 0.0000 | 0.0000 |
| f__Ginkgoaceae;g__Ginkgo;s__*Ginkgo biloba* | 0.0000 | 0.0000 | 0.0041 | 0.0000 | 0.0000 | 0.0000 |
| f__Poaceae;g__Sporobolus;s__*Sporobolus aculeatus* | 0.0000 | 0.0000 | 0.0040 | 0.0000 | 0.0000 | 0.0000 |
| f__Cornaceae;g__Cornus;s__*Cornus amomum* | 0.0005 | 0.0026 | 0.0005 | 0.0001 | 0.0000 | 0.0000 |
| f__Brassicaceae;g__Brassica;s__*Brassica rapa subsp. rapa* | 0.0000 | 0.0000 | 0.0000 | 0.0012 | 0.0000 | 0.0003 |
| f__Musaceae;g__Musa;s__*Musa hybrid cultivar* | 0.0000 | 0.0000 | 0.0000 | 0.0000 | 0.0000 | 0.0000 |
| f__Fabaceae;g__Pterocarpus;s__*Pterocarpus mildbraedii* | 0.0001 | 0.0000 | 0.0000 | 0.0000 | 0.0000 | 0.0000 |
| f__Cucurbitaceae;g__Corallocarpus;s__*Corallocarpus bainesii* | 0.0000 | 0.0000 | 0.0000 | 0.0000 | 0.0000 | 0.0000 |
| f__Caryophyllaceae;g__Cerastium;s__*Cerastium nutans* | 0.0001 | 0.0000 | 0.0002 | 0.0007 | 0.0000 | 0.0000 |
| f__Poaceae;g__Oryza;s__*Oryza meridionalis* | 0.0000 | 0.0000 | 0.0000 | 0.0001 | 0.0004 | 0.0000 |
| f__Rutaceae;g__Zanthoxylum;s__*Zanthoxylum simulans* | 0.0000 | 0.0000 | 0.0000 | 0.0000 | 0.0000 | 0.0000 |
| f__Juglandaceae;g__Cyclocarya;s__*Cyclocarya paliurus* | 0.0002 | 0.0000 | 0.0000 | 0.0000 | 0.0004 | 0.0000 |
| f__Oxalidaceae;g__Oxalis;s__*Oxalis oregana* | 0.0000 | 0.0018 | 0.0000 | 0.0000 | 0.0000 | 0.0000 |
| f__Lauraceae;g__Cryptocarya;s__*Cryptocarya bidwillii* | 0.0000 | 0.0000 | 0.0001 | 0.0002 | 0.0003 | 0.0002 |
| f__Corynocarpaceae;g__Corynocarpus;s__*Corynocarpus rupestris* | 0.0001 | 0.0000 | 0.0000 | 0.0000 | 0.0000 | 0.0000 |
| f__Rutaceae;g__Zanthoxylum;s__*Zanthoxylum americanum* | 0.0000 | 0.0000 | 0.0000 | 0.0000 | 0.0000 | 0.0000 |
| f__Musaceae;g__Ensete;s__*Ensete ventricosum* | 0.0000 | 0.0000 | 0.0000 | 0.0000 | 0.0000 | 0.0000 |
| f__Polygonaceae;g__Bistorta;s__*Bistorta bistortoides* | 0.0000 | 0.0000 | 0.0000 | 0.0000 | 0.0000 | 0.0000 |
| f__Solanaceae;g__Solanum;s__*Solanum giganteum* | 0.0000 | 0.0002 | 0.0000 | 0.0000 | 0.0000 | 0.0000 |
| f__Cornaceae;g__Cornus;s__*Cornus racemosa* | 0.0007 | 0.0002 | 0.0000 | 0.0000 | 0.0000 | 0.0000 |
| f__Euphorbiaceae;g__Acalypha;s__*Acalypha rhomboidea* | 0.0000 | 0.0000 | 0.0000 | 0.0000 | 0.0000 | 0.0000 |
| f__Fabaceae;g__Pterocarpus;s__*Pterocarpus soyauxii* | 0.0000 | 0.0000 | 0.0000 | 0.0000 | 0.0000 | 0.0000 |
| f__Cucurbitaceae;g__Cucumis;s__*Cucumis melo* | 0.0000 | 0.0000 | 0.0000 | 0.0000 | 0.0000 | 0.0000 |
| f__Pylaisiadelphaceae;g__Brotherella;s__*Brotherella recurvans* | 0.0006 | 0.0000 | 0.0000 | 0.0000 | 0.0000 | 0.0000 |
| f__Cucurbitaceae;g__Thladiantha;s__*Thladiantha villosula* | 0.0001 | 0.0000 | 0.0000 | 0.0000 | 0.0000 | 0.0000 |
| f__Corynocarpaceae;g__Corynocarpus;s__*Corynocarpus laevigatus* | 0.0000 | 0.0000 | 0.0000 | 0.0000 | 0.0000 | 0.0000 |
| f__Lauraceae;g__Cinnamomum;s__*Cinnamomum aromaticum* | 0.0001 | 0.0002 | 0.0001 | 0.0000 | 0.0000 | 0.0000 |
| f__Rosaceae;g__Prunus;s__*Prunus longistyla* | 0.0003 | 0.0000 | 0.0001 | 0.0000 | 0.0000 | 0.0000 |
| f__Fabaceae;g__Medicago;s__*Medicago rotata* | 0.0000 | 0.0000 | 0.0000 | 0.0000 | 0.0000 | 0.0000 |
| f__Caryophyllaceae;g__Cerastium;s__*Cerastium tianschanicum* | 0.0000 | 0.0000 | 0.0000 | 0.0000 | 0.0000 | 0.0000 |
| f__Euphorbiaceae;g__Cnidoscolus;s__*Cnidoscolus urens* | 0.0000 | 0.0000 | 0.0000 | 0.0000 | 0.0000 | 0.0000 |
| f__Cucurbitaceae;g__Melothria;s__*Melothria trilobata* | 0.0000 | 0.0000 | 0.0000 | 0.0000 | 0.0000 | 0.0000 |
| f__Poaceae;g__Oryza;s__*Oryza ridleyi* | 0.0000 | 0.0000 | 0.0000 | 0.0000 | 0.0002 | 0.0000 |
| f__Solanaceae;g__Solanum;s__*Solanum tuberosum* | 0.0000 | 0.0000 | 0.0000 | 0.0000 | 0.0000 | 0.0000 |
| Supplementary Table 2.2 Relative abundance of plant food items at the species level in all Chinese mole shrew diet samples during summer. | | | | | | |
| Taxon | Relative abundance (Summer) | | | | | |
|  | Su1560a | Su1560b | Su1560c | Su1568a | Su1568b | Su1568c |
| f__Fabaceae;g__Arachis;s__*Arachis hypogaea* | 0.2457 | 0.0000 | 0.0663 | 0.0000 | 0.0001 | 0.2727 |
| f__Poaceae;g__Chikusichloa;s__*Chikusichloa aquatica* | 0.0000 | 0.0001 | 0.0000 | 0.0000 | 0.0000 | 0.0000 |
| f__Lauraceae;g__Cinnamomum;s__*Cinnamomum glaucescens* | 0.0001 | 0.0000 | 0.0002 | 0.0000 | 0.0021 | 0.0000 |
| f__Caryophyllaceae;g__Cerastium;s__*Cerastium glomeratum* | 0.0001 | 0.0000 | 0.0000 | 0.0000 | 0.0000 | 0.0000 |
| f__Oleaceae;g__Nestegis;s__*Nestegis apetala* | 0.4104 | 0.4437 | 0.2237 | 0.0195 | 0.0000 | 0.0000 |
| f__unidentified;g__Camptotheca;s__*Camptotheca acuminata* | 0.0003 | 0.0001 | 0.0058 | 0.0042 | 0.4370 | 0.1908 |
| f__Caryophyllaceae;g__Stellaria;s__*Stellaria media* | 0.0000 | 0.0000 | 0.0000 | 0.0000 | 0.0000 | 0.0000 |
| f__Phytolaccaceae;g__Phytolacca;s__*Phytolacca americana* | 0.0000 | 0.0000 | 0.0000 | 0.0000 | 0.0000 | 0.0000 |
| f__Poaceae;g__Oryza;s__*Oryza sativa* | 0.0000 | 0.0048 | 0.0000 | 0.0000 | 0.0000 | 0.0000 |
| f__Cyperaceae;g__Carex;s__*Carex aneurocarpa* | 0.3366 | 0.3376 | 0.0026 | 0.0000 | 0.0000 | 0.0000 |
| f__Iridaceae;g__Gladiolus;s__*Gladiolus x gandavensis* | 0.0000 | 0.0000 | 0.0000 | 0.0000 | 0.0000 | 0.0000 |
| f__Solanaceae;g__Withania;s__*Withania frutescens* | 0.0000 | 0.0000 | 0.0000 | 0.4771 | 0.0001 | 0.0000 |
| f__Musaceae;g__Musa;s__*Musa laterita* | 0.0002 | 0.1678 | 0.2285 | 0.0000 | 0.0000 | 0.0000 |
| f__Lauraceae;g__Cinnamomum;s__*Cinnamomum bodinieri* | 0.0000 | 0.0000 | 0.0000 | 0.0000 | 0.0000 | 0.0000 |
| f__Mazaceae;g__Mazus;s__*Mazus reptans* | 0.0000 | 0.0000 | 0.0000 | 0.0000 | 0.0000 | 0.0000 |
| f__Asteraceae;g__Lactuca;s__*Lactuca sativa* | 0.0000 | 0.0000 | 0.0014 | 0.0000 | 0.0000 | 0.0000 |
| f__Asteraceae;g__Galinsoga;s__*Galinsoga parviflora* | 0.0000 | 0.0000 | 0.0002 | 0.1123 | 0.1563 | 0.0778 |
| f__Ranunculaceae;g__Isopyrum;s__*Isopyrum biternatum* | 0.0000 | 0.0000 | 0.0000 | 0.0000 | 0.0001 | 0.3002 |
| f__Rosaceae;g__Prunus;s__*Prunus mongolica* | 0.0000 | 0.0000 | 0.0371 | 0.0002 | 0.2806 | 0.0001 |
| f__Pedaliaceae;g__Uncarina;s__*Uncarina grandidieri* | 0.0004 | 0.0004 | 0.0049 | 0.2912 | 0.0000 | 0.0000 |
| f__Fabaceae;g__Cyamopsis;s__*Cyamopsis tetragonoloba* | 0.0000 | 0.0000 | 0.0000 | 0.0000 | 0.0000 | 0.0000 |
| f__Linderniaceae;g__Lindernia;s__*Lindernia antipoda* | 0.0000 | 0.0000 | 0.0000 | 0.0000 | 0.0000 | 0.0000 |
| f__Asteraceae;g__Leontopodium;s__*Leontopodium alpinum* | 0.0000 | 0.0000 | 0.0001 | 0.0000 | 0.0982 | 0.1227 |
| f__Poaceae;g__Eleusine;s__*Eleusine indica* | 0.0000 | 0.0000 | 0.0000 | 0.0000 | 0.0000 | 0.0000 |
| f__Polygonaceae;g__Bistorta;s__*Bistorta vivipara* | 0.0001 | 0.0000 | 0.1746 | 0.0000 | 0.0000 | 0.0000 |
| f__Juglandaceae;g__Carya;s__*Carya glabra* | 0.0000 | 0.0000 | 0.0983 | 0.0000 | 0.0000 | 0.0000 |
| f__Poaceae;g__Orinus;s__*Orinus kokonoricus* | 0.0000 | 0.0000 | 0.0000 | 0.0000 | 0.0000 | 0.0000 |
| f__Fabaceae;g__Medicago;s__*Medicago sativa* | 0.0000 | 0.0000 | 0.0000 | 0.0000 | 0.0000 | 0.0000 |
| f__Alismataceae;g__Sagittaria;s__*Sagittaria trifolia* | 0.0000 | 0.0000 | 0.0000 | 0.0000 | 0.0000 | 0.0000 |
| f__Amaryllidaceae;g__Allium;s__*Allium stellatum* | 0.0000 | 0.0000 | 0.0000 | 0.0000 | 0.0000 | 0.0000 |
| f__Euphorbiaceae;g__Euphorbia;s__*Euphorbia hypericifolia* | 0.0000 | 0.0000 | 0.0000 | 0.0000 | 0.0000 | 0.0000 |
| f__Amaryllidaceae;g__Allium;s__*Allium prattii* | 0.0000 | 0.0000 | 0.0000 | 0.0000 | 0.0000 | 0.0000 |
| f__Euphorbiaceae;g__Acalypha;s__*Acalypha australis* | 0.0000 | 0.0000 | 0.0000 | 0.0000 | 0.0000 | 0.0000 |
| f__Asteraceae;g__Faberia;s__*Faberia pinnatifida* | 0.0000 | 0.0000 | 0.0399 | 0.0000 | 0.0000 | 0.0000 |
| f__Pylaisiadelphaceae;g__Pylaisiadelpha;s__*Pylaisiadelpha tenuirostris* | 0.0000 | 0.0000 | 0.0000 | 0.0000 | 0.0000 | 0.0000 |
| f__Apiaceae;g__Ligusticum;s__*Ligusticum sinense* | 0.0000 | 0.0000 | 0.0000 | 0.0000 | 0.0000 | 0.0003 |
| f__Rubiaceae;g__Uncaria;s__*Uncaria tomentosa* | 0.0000 | 0.0000 | 0.0000 | 0.0000 | 0.0000 | 0.0000 |
| f__Hypnaceae;g__Callicladium;s__*Callicladium haldanianum* | 0.0000 | 0.0000 | 0.0000 | 0.0000 | 0.0000 | 0.0000 |
| f__Fabaceae;g__Medicago;s__*Medicago laciniata* | 0.0000 | 0.0000 | 0.0000 | 0.0000 | 0.0000 | 0.0000 |
| f__Rosaceae;g__Rosa;s__*Rosa chinensis var. spontanea* | 0.0000 | 0.0000 | 0.0589 | 0.0000 | 0.0000 | 0.0000 |
| f__Rubiaceae;g__Ophiorrhiza;s__*Ophiorrhiza sp. SH-2010* | 0.0037 | 0.0013 | 0.0016 | 0.0001 | 0.0000 | 0.0000 |
| f__Oleaceae;g__Osmanthus;s__*Osmanthus americanus* | 0.0019 | 0.0211 | 0.0206 | 0.0075 | 0.0000 | 0.0000 |
| f__Polygonaceae;g__Rumex;s__*Rumex utahensis* | 0.0000 | 0.0000 | 0.0000 | 0.0000 | 0.0000 | 0.0000 |
| f__Musaceae;g__Musa;s__*Musa acuminata* | 0.0000 | 0.0226 | 0.0234 | 0.0000 | 0.0000 | 0.0000 |
| f__Moraceae;g__Ficus;s__*Ficus benghalensis* | 0.0000 | 0.0000 | 0.0000 | 0.0000 | 0.0000 | 0.0000 |
| f__Cucurbitaceae;g__Cucurbita;s__*Cucurbita pepo* | 0.0000 | 0.0000 | 0.0000 | 0.0442 | 0.0000 | 0.0000 |
| f__Convolvulaceae;g__Convolvulus;s__*Convolvulus arvensis* | 0.0000 | 0.0000 | 0.0000 | 0.0000 | 0.0000 | 0.0000 |
| f__Poaceae;g__Oryza;s__*Oryza rhizomatis* | 0.0000 | 0.0001 | 0.0000 | 0.0000 | 0.0000 | 0.0000 |
| f__Brassicaceae;g__Brassica;s__*Brassica oleracea var. botrytis* | 0.0000 | 0.0000 | 0.0001 | 0.0000 | 0.0000 | 0.0000 |
| f__Asteraceae;g__Helichrysum;s__*Helichrysum zeyheri* | 0.0000 | 0.0000 | 0.0011 | 0.0025 | 0.0202 | 0.0149 |
| f__Araceae;g__Lemna;s__*Lemna minor* | 0.0000 | 0.0000 | 0.0000 | 0.0000 | 0.0000 | 0.0000 |
| f__Cornaceae;g__Cornus;s__*Cornus sericea* | 0.0000 | 0.0000 | 0.0000 | 0.0000 | 0.0000 | 0.0000 |
| f__Betulaceae;g__Alnus;s__*Alnus rubra* | 0.0000 | 0.0000 | 0.0000 | 0.0000 | 0.0000 | 0.0000 |
| f__Solanaceae;g__Capsicum;s__*Capsicum annuum* | 0.0000 | 0.0000 | 0.0000 | 0.0232 | 0.0000 | 0.0000 |
| f__Iridaceae;g__Gladiolus;s__*Gladiolus palustris* | 0.0000 | 0.0000 | 0.0000 | 0.0000 | 0.0000 | 0.0000 |
| f__Cucurbitaceae;g__Cucumis;s__*Cucumis sativus* | 0.0000 | 0.0000 | 0.0000 | 0.0019 | 0.0000 | 0.0000 |
| f__Cucurbitaceae;g__Cucurbita;s__*Cucurbita moschata* | 0.0000 | 0.0000 | 0.0000 | 0.0037 | 0.0000 | 0.0000 |
| f__Fabaceae;g__Glycine;s__*Glycine canescens* | 0.0000 | 0.0000 | 0.0000 | 0.0000 | 0.0000 | 0.0000 |
| f__Oxalidaceae;g__Oxalis;s__*Oxalis debilis* | 0.0000 | 0.0000 | 0.0000 | 0.0000 | 0.0000 | 0.0000 |
| f__Fabaceae;g__Kummerowia;s__*Kummerowia striata* | 0.0000 | 0.0000 | 0.0000 | 0.0000 | 0.0000 | 0.0000 |
| f__Fabaceae;g__Stylosanthes;s__*Stylosanthes viscosa* | 0.0004 | 0.0000 | 0.0003 | 0.0000 | 0.0000 | 0.0002 |
| f__Achatocarpaceae;g__Achatocarpus;s__*Achatocarpus gracilis* | 0.0000 | 0.0000 | 0.0000 | 0.0000 | 0.0000 | 0.0000 |
| f__Poaceae;g__Oryza;s__*Oryza longiglumis* | 0.0000 | 0.0000 | 0.0000 | 0.0000 | 0.0000 | 0.0000 |
| f__Fabaceae;g__Pterocarpus;s__*Pterocarpus ternatus* | 0.0000 | 0.0000 | 0.0001 | 0.0000 | 0.0000 | 0.0079 |
| f__Solanaceae;g__Lycium;s__*Lycium chinense* | 0.0000 | 0.0000 | 0.0000 | 0.0038 | 0.0000 | 0.0000 |
| f__Ranunculaceae;g__Isopyrum;s__*Isopyrum savilei* | 0.0000 | 0.0000 | 0.0000 | 0.0000 | 0.0000 | 0.0118 |
| f__Apiaceae;g__Chaerophyllum;s__*Chaerophyllum procumbens* | 0.0000 | 0.0000 | 0.0000 | 0.0000 | 0.0000 | 0.0000 |
| f__Brassicaceae;g__Draba;s__*Draba densifolia* | 0.0000 | 0.0000 | 0.0000 | 0.0000 | 0.0000 | 0.0000 |
| f__Caryophyllaceae;g__Cerastium;s__*Cerastium arvense* | 0.0000 | 0.0000 | 0.0000 | 0.0000 | 0.0000 | 0.0000 |
| f__Fabaceae;g__Craspedolobium;s__*Craspedolobium unijugum* | 0.0000 | 0.0000 | 0.0000 | 0.0000 | 0.0000 | 0.0006 |
| f__Caryophyllaceae;g__Stellaria;s__*Stellaria holostea* | 0.0000 | 0.0000 | 0.0000 | 0.0000 | 0.0000 | 0.0000 |
| f__Solanaceae;g__Solanum;s__*Solanum sp. Espinoza*5665 | 0.0000 | 0.0000 | 0.0000 | 0.0034 | 0.0000 | 0.0000 |
| f__Rosaceae;g__Prunus;s__*Prunus takesimensis* | 0.0000 | 0.0000 | 0.0007 | 0.0000 | 0.0053 | 0.0000 |
| f__Caryophyllaceae;g__Stellaria;s__*Stellaria americana* | 0.0000 | 0.0000 | 0.0000 | 0.0000 | 0.0000 | 0.0000 |
| f__Poaceae;g__Ctenium;s__*Ctenium floridanum* | 0.0000 | 0.0000 | 0.0000 | 0.0000 | 0.0000 | 0.0000 |
| f__Oxalidaceae;g__Oxalis;s__*Oxalis violacea* | 0.0000 | 0.0000 | 0.0000 | 0.0000 | 0.0000 | 0.0000 |
| f__Amaranthaceae;g__Achyranthes;s__*Achyranthes aspera* | 0.0000 | 0.0000 | 0.0000 | 0.0000 | 0.0000 | 0.0000 |
| f__Cucurbitaceae;g__Luffa;s__*Luffa quinquefida* | 0.0000 | 0.0000 | 0.0000 | 0.0047 | 0.0000 | 0.0000 |
| f__Rutaceae;g__Zanthoxylum;s__*Zanthoxylum clava-herculis* | 0.0000 | 0.0000 | 0.0000 | 0.0000 | 0.0000 | 0.0000 |
| f__Polygonaceae;g__Bistorta;s__*Bistorta officinalis* | 0.0000 | 0.0000 | 0.0045 | 0.0000 | 0.0000 | 0.0000 |
| f__Ginkgoaceae;g__Ginkgo;s__*Ginkgo biloba* | 0.0000 | 0.0000 | 0.0000 | 0.0000 | 0.0000 | 0.0000 |
| f__Poaceae;g__Sporobolus;s__*Sporobolus aculeatus* | 0.0000 | 0.0000 | 0.0000 | 0.0000 | 0.0000 | 0.0000 |
| f__Cornaceae;g__Cornus;s__*Cornus amomum* | 0.0000 | 0.0000 | 0.0000 | 0.0000 | 0.0000 | 0.0000 |
| f__Brassicaceae;g__Brassica;s__*Brassica rapa subsp. rapa* | 0.0000 | 0.0000 | 0.0000 | 0.0000 | 0.0000 | 0.0000 |
| f__Musaceae;g__Musa;s__*Musa hybrid cultivar* | 0.0000 | 0.0000 | 0.0020 | 0.0000 | 0.0000 | 0.0000 |
| f__Fabaceae;g__Pterocarpus;s__*Pterocarpus mildbraedii* | 0.0000 | 0.0000 | 0.0000 | 0.0000 | 0.0000 | 0.0000 |
| f__Cucurbitaceae;g__Corallocarpus;s__*Corallocarpus bainesii* | 0.0000 | 0.0000 | 0.0000 | 0.0000 | 0.0000 | 0.0000 |
| f__Caryophyllaceae;g__Cerastium;s__*Cerastium nutans* | 0.0000 | 0.0000 | 0.0000 | 0.0000 | 0.0000 | 0.0000 |
| f__Poaceae;g__Oryza;s__*Oryza meridionalis* | 0.0000 | 0.0000 | 0.0000 | 0.0000 | 0.0000 | 0.0000 |
| f__Rutaceae;g__Zanthoxylum;s__*Zanthoxylum simulans* | 0.0000 | 0.0000 | 0.0000 | 0.0000 | 0.0000 | 0.0000 |
| f__Juglandaceae;g__Cyclocarya;s__*Cyclocarya paliurus* | 0.0000 | 0.0000 | 0.0013 | 0.0000 | 0.0000 | 0.0000 |
| f__Oxalidaceae;g__Oxalis;s__*Oxalis oregana* | 0.0000 | 0.0000 | 0.0000 | 0.0000 | 0.0000 | 0.0000 |
| f__Lauraceae;g__Cryptocarya;s__*Cryptocarya bidwillii* | 0.0000 | 0.0000 | 0.0000 | 0.0000 | 0.0000 | 0.0000 |
| f__Corynocarpaceae;g__Corynocarpus;s__*Corynocarpus rupestris* | 0.0000 | 0.0000 | 0.0000 | 0.0000 | 0.0000 | 0.0000 |
| f__Rutaceae;g__Zanthoxylum;s__*Zanthoxylum americanum* | 0.0000 | 0.0000 | 0.0000 | 0.0000 | 0.0000 | 0.0000 |
| f__Musaceae;g__Ensete;s__*Ensete ventricosum* | 0.0000 | 0.0002 | 0.0009 | 0.0000 | 0.0000 | 0.0000 |
| f__Polygonaceae;g__Bistorta;s__*Bistorta bistortoides* | 0.0000 | 0.0000 | 0.0010 | 0.0000 | 0.0000 | 0.0000 |
| f__Solanaceae;g__Solanum;s__*Solanum giganteum* | 0.0000 | 0.0000 | 0.0000 | 0.0005 | 0.0000 | 0.0000 |
| f__Cornaceae;g__Cornus;s__*Cornus racemosa* | 0.0000 | 0.0000 | 0.0000 | 0.0000 | 0.0000 | 0.0000 |
| f__Euphorbiaceae;g__Acalypha;s__*Acalypha rhomboidea* | 0.0000 | 0.0000 | 0.0000 | 0.0000 | 0.0000 | 0.0000 |
| f__Fabaceae;g__Pterocarpus;s__*Pterocarpus soyauxii* | 0.0000 | 0.0000 | 0.0000 | 0.0000 | 0.0000 | 0.0000 |
| f__Cucurbitaceae;g__Cucumis;s__*Cucumis melo* | 0.0000 | 0.0000 | 0.0000 | 0.0000 | 0.0000 | 0.0000 |
| f__Pylaisiadelphaceae;g__Brotherella;s__*Brotherella recurvans* | 0.0000 | 0.0000 | 0.0000 | 0.0000 | 0.0000 | 0.0000 |
| f__Cucurbitaceae;g__Thladiantha;s__*Thladiantha villosula* | 0.0000 | 0.0000 | 0.0000 | 0.0000 | 0.0000 | 0.0000 |
| f__Corynocarpaceae;g__Corynocarpus;s__*Corynocarpus laevigatus* | 0.0000 | 0.0000 | 0.0000 | 0.0000 | 0.0000 | 0.0000 |
| f__Lauraceae;g__Cinnamomum;s__*Cinnamomum aromaticum* | 0.0000 | 0.0000 | 0.0000 | 0.0000 | 0.0000 | 0.0000 |
| f__Rosaceae;g__Prunus;s__*Prunus longistyla* | 0.0000 | 0.0000 | 0.0000 | 0.0000 | 0.0000 | 0.0000 |
| f__Fabaceae;g__Medicago;s__*Medicago rotata* | 0.0000 | 0.0000 | 0.0000 | 0.0000 | 0.0000 | 0.0000 |
| f__Caryophyllaceae;g__Cerastium;s__*Cerastium tianschanicum* | 0.0000 | 0.0000 | 0.0000 | 0.0000 | 0.0000 | 0.0000 |
| f__Euphorbiaceae;g__Cnidoscolus;s__*Cnidoscolus urens* | 0.0000 | 0.0000 | 0.0000 | 0.0000 | 0.0000 | 0.0000 |
| f__Cucurbitaceae;g__Melothria;s__*Melothria trilobata* | 0.0000 | 0.0000 | 0.0000 | 0.0001 | 0.0000 | 0.0000 |
| f__Poaceae;g__Oryza;s__*Oryza ridleyi* | 0.0000 | 0.0000 | 0.0000 | 0.0000 | 0.0000 | 0.0000 |
| f__Solanaceae;g__Solanum;s__*Solanum tuberosum* | 0.0000 | 0.0000 | 0.0000 | 0.0000 | 0.0000 | 0.0000 |
| Supplementary Table 2.3 Relative abundance of plant food items at the species level in all Chinese mole shrew diet samples during autumn. | | | | | | |
| Taxon | Relative abundance (Autumn) | | | | | |
|  | A1003a | A1003b | A1003c | A1011a | A1011b | A1011c |
| f__Fabaceae;g__Arachis;s__*Arachis hypogaea* | 0.0000 | 0.0002 | 0.0001 | 0.0918 | 0.0562 | 0.0508 |
| f__Poaceae;g__Chikusichloa;s__*Chikusichloa aquatica* | 0.3613 | 0.3400 | 0.2309 | 0.2668 | 0.4434 | 0.5423 |
| f__Lauraceae;g__Cinnamomum;s__*Cinnamomum glaucescens* | 0.0000 | 0.0001 | 0.0001 | 0.0003 | 0.0003 | 0.0009 |
| f__Caryophyllaceae;g__Cerastium;s__*Cerastium glomeratum* | 0.0000 | 0.0000 | 0.0000 | 0.0000 | 0.0000 | 0.0000 |
| f__Oleaceae;g__Nestegis;s__*Nestegis apetala* | 0.0000 | 0.0000 | 0.0000 | 0.0000 | 0.0003 | 0.0000 |
| f__unidentified;g__Camptotheca;s__*Camptotheca acuminata* | 0.0001 | 0.0000 | 0.0003 | 0.2302 | 0.1311 | 0.0750 |
| f__Caryophyllaceae;g__Stellaria;s__*Stellaria media* | 0.0000 | 0.0000 | 0.0000 | 0.0000 | 0.0000 | 0.0000 |
| f__Phytolaccaceae;g__Phytolacca;s__*Phytolacca americana* | 0.4138 | 0.2539 | 0.3147 | 0.0004 | 0.0001 | 0.0000 |
| f__Poaceae;g__Oryza;s__*Oryza sativa* | 0.0131 | 0.2605 | 0.0736 | 0.0252 | 0.1860 | 0.1187 |
| f__Cyperaceae;g__Carex;s__*Carex aneurocarpa* | 0.0000 | 0.0000 | 0.0000 | 0.0000 | 0.0000 | 0.0000 |
| f__Iridaceae;g__Gladiolus;s__*Gladiolus x gandavensis* | 0.0000 | 0.0000 | 0.0000 | 0.0000 | 0.0000 | 0.0000 |
| f__Solanaceae;g__Withania;s__*Withania frutescens* | 0.0531 | 0.0000 | 0.0000 | 0.0060 | 0.0128 | 0.0000 |
| f__Musaceae;g__Musa;s__*Musa laterita* | 0.0000 | 0.0000 | 0.0000 | 0.0017 | 0.0000 | 0.0000 |
| f__Lauraceae;g__Cinnamomum;s__*Cinnamomum bodinieri* | 0.0000 | 0.0740 | 0.0078 | 0.0031 | 0.0001 | 0.0000 |
| f__Mazaceae;g__Mazus;s__*Mazus reptans* | 0.0000 | 0.0000 | 0.0000 | 0.0000 | 0.0000 | 0.0000 |
| f__Asteraceae;g__Lactuca;s__*Lactuca sativa* | 0.0000 | 0.0000 | 0.0000 | 0.0148 | 0.0001 | 0.0125 |
| f__Asteraceae;g__Galinsoga;s__*Galinsoga parviflora* | 0.0062 | 0.0000 | 0.0000 | 0.0077 | 0.0033 | 0.0000 |
| f__Ranunculaceae;g__Isopyrum;s__*Isopyrum biternatum* | 0.0000 | 0.0000 | 0.0000 | 0.0298 | 0.0000 | 0.0000 |
| f__Rosaceae;g__Prunus;s__*Prunus mongolica* | 0.0000 | 0.0000 | 0.0000 | 0.0011 | 0.0000 | 0.0000 |
| f__Pedaliaceae;g__Uncarina;s__*Uncarina grandidieri* | 0.0000 | 0.0000 | 0.0000 | 0.0052 | 0.0005 | 0.0000 |
| f__Fabaceae;g__Cyamopsis;s__*Cyamopsis tetragonoloba* | 0.0000 | 0.0000 | 0.0000 | 0.0061 | 0.0007 | 0.0000 |
| f__Linderniaceae;g__Lindernia;s__*Lindernia antipoda* | 0.0000 | 0.0000 | 0.0000 | 0.1171 | 0.0662 | 0.0804 |
| f__Asteraceae;g__Leontopodium;s__*Leontopodium alpinum* | 0.0000 | 0.0000 | 0.0000 | 0.0121 | 0.0000 | 0.0003 |
| f__Poaceae;g__Eleusine;s__*Eleusine indica* | 0.0139 | 0.0248 | 0.1911 | 0.0000 | 0.0004 | 0.0000 |
| f__Polygonaceae;g__Bistorta;s__*Bistorta vivipara* | 0.0000 | 0.0000 | 0.0000 | 0.0000 | 0.0000 | 0.0000 |
| f__Juglandaceae;g__Carya;s__*Carya glabra* | 0.0000 | 0.0000 | 0.0000 | 0.0000 | 0.0000 | 0.0000 |
| f__Poaceae;g__Orinus;s__*Orinus kokonoricus* | 0.0000 | 0.0003 | 0.0015 | 0.0001 | 0.0000 | 0.0000 |
| f__Fabaceae;g__Medicago;s__*Medicago sativa* | 0.0053 | 0.0069 | 0.0809 | 0.0015 | 0.0000 | 0.0544 |
| f__Alismataceae;g__Sagittaria;s__*Sagittaria trifolia* | 0.0000 | 0.0000 | 0.0000 | 0.0000 | 0.0076 | 0.0000 |
| f__Amaryllidaceae;g__Allium;s__*Allium stellatum* | 0.0000 | 0.0000 | 0.0000 | 0.0000 | 0.0000 | 0.0000 |
| f__Euphorbiaceae;g__Euphorbia;s__*Euphorbia hypericifolia* | 0.0162 | 0.0001 | 0.0002 | 0.0000 | 0.0000 | 0.0001 |
| f__Amaryllidaceae;g__Allium;s__*Allium prattii* | 0.0000 | 0.0000 | 0.0410 | 0.0000 | 0.0000 | 0.0000 |
| f__Euphorbiaceae;g__Acalypha;s__*Acalypha australis* | 0.0391 | 0.0068 | 0.0102 | 0.0000 | 0.0000 | 0.0003 |
| f__Asteraceae;g__Faberia;s__*Faberia pinnatifida* | 0.0000 | 0.0000 | 0.0000 | 0.0000 | 0.0000 | 0.0002 |
| f__Pylaisiadelphaceae;g__Pylaisiadelpha;s__*Pylaisiadelpha tenuirostris* | 0.0000 | 0.0000 | 0.0000 | 0.0000 | 0.0000 | 0.0000 |
| f__Apiaceae;g__Ligusticum;s__*Ligusticum sinense* | 0.0000 | 0.0000 | 0.0000 | 0.0000 | 0.0511 | 0.0235 |
| f__Rubiaceae;g__Uncaria;s__*Uncaria tomentosa* | 0.0755 | 0.0000 | 0.0000 | 0.0001 | 0.0001 | 0.0001 |
| f__Hypnaceae;g__Callicladium;s__*Callicladium haldanianum* | 0.0000 | 0.0000 | 0.0000 | 0.0729 | 0.0000 | 0.0000 |
| f__Fabaceae;g__Medicago;s__*Medicago laciniata* | 0.0000 | 0.0000 | 0.0002 | 0.0000 | 0.0000 | 0.0001 |
| f__Rosaceae;g__Rosa;s__*Rosa chinensis var. spontanea* | 0.0000 | 0.0000 | 0.0000 | 0.0000 | 0.0000 | 0.0000 |
| f__Rubiaceae;g__Ophiorrhiza;s__*Ophiorrhiza sp. SH-2010* | 0.0000 | 0.0000 | 0.0000 | 0.0000 | 0.0002 | 0.0000 |
| f__Oleaceae;g__Osmanthus;s__*Osmanthus americanus* | 0.0000 | 0.0000 | 0.0000 | 0.0000 | 0.0001 | 0.0000 |
| f__Polygonaceae;g__Rumex;s__*Rumex utahensis* | 0.0002 | 0.0000 | 0.0000 | 0.0000 | 0.0000 | 0.0000 |
| f__Musaceae;g__Musa;s__*Musa acuminata* | 0.0000 | 0.0000 | 0.0000 | 0.0000 | 0.0000 | 0.0000 |
| f__Moraceae;g__Ficus;s__*Ficus benghalensis* | 0.0000 | 0.0000 | 0.0000 | 0.0448 | 0.0000 | 0.0021 |
| f__Cucurbitaceae;g__Cucurbita;s__*Cucurbita pepo* | 0.0000 | 0.0000 | 0.0000 | 0.0006 | 0.0004 | 0.0005 |
| f__Convolvulaceae;g__Convolvulus;s__*Convolvulus arvensis* | 0.0001 | 0.0023 | 0.0402 | 0.0001 | 0.0003 | 0.0011 |
| f__Poaceae;g__Oryza;s__*Oryza rhizomatis* | 0.0010 | 0.0271 | 0.0000 | 0.0001 | 0.0101 | 0.0040 |
| f__Brassicaceae;g__Brassica;s__*Brassica oleracea var. botrytis* | 0.0000 | 0.0000 | 0.0000 | 0.0000 | 0.0065 | 0.0061 |
| f__Asteraceae;g__Helichrysum;s__*Helichrysum zeyheri* | 0.0000 | 0.0000 | 0.0000 | 0.0014 | 0.0002 | 0.0007 |
| f__Araceae;g__Lemna;s__*Lemna minor* | 0.0000 | 0.0000 | 0.0000 | 0.0384 | 0.0000 | 0.0044 |
| f__Cornaceae;g__Cornus;s__*Cornus sericea* | 0.0000 | 0.0000 | 0.0000 | 0.0000 | 0.0000 | 0.0000 |
| f__Betulaceae;g__Alnus;s__*Alnus rubra* | 0.0000 | 0.0000 | 0.0000 | 0.0000 | 0.0000 | 0.0000 |
| f__Solanaceae;g__Capsicum;s__*Capsicum annuum* | 0.0006 | 0.0000 | 0.0000 | 0.0002 | 0.0030 | 0.0000 |
| f__Iridaceae;g__Gladiolus;s__*Gladiolus palustris* | 0.0000 | 0.0000 | 0.0000 | 0.0000 | 0.0000 | 0.0000 |
| f__Cucurbitaceae;g__Cucumis;s__*Cucumis sativus* | 0.0000 | 0.0000 | 0.0000 | 0.0002 | 0.0066 | 0.0127 |
| f__Cucurbitaceae;g__Cucurbita;s__*Cucurbita moschata* | 0.0000 | 0.0000 | 0.0000 | 0.0181 | 0.0000 | 0.0002 |
| f__Fabaceae;g__Glycine;s__*Glycine canescens* | 0.0000 | 0.0000 | 0.0000 | 0.0000 | 0.0001 | 0.0000 |
| f__Oxalidaceae;g__Oxalis;s__*Oxalis debilis* | 0.0000 | 0.0000 | 0.0000 | 0.0000 | 0.0000 | 0.0000 |
| f__Fabaceae;g__Kummerowia;s__*Kummerowia striata* | 0.0000 | 0.0000 | 0.0000 | 0.0000 | 0.0001 | 0.0001 |
| f__Fabaceae;g__Stylosanthes;s__*Stylosanthes viscosa* | 0.0000 | 0.0000 | 0.0000 | 0.0001 | 0.0000 | 0.0001 |
| f__Achatocarpaceae;g__Achatocarpus;s__*Achatocarpus gracilis* | 0.0000 | 0.0000 | 0.0000 | 0.0000 | 0.0002 | 0.0000 |
| f__Poaceae;g__Oryza;s__*Oryza longiglumis* | 0.0003 | 0.0029 | 0.0066 | 0.0000 | 0.0022 | 0.0016 |
| f__Fabaceae;g__Pterocarpus;s__*Pterocarpus ternatus* | 0.0000 | 0.0000 | 0.0000 | 0.0000 | 0.0021 | 0.0012 |
| f__Solanaceae;g__Lycium;s__*Lycium chinense* | 0.0000 | 0.0000 | 0.0000 | 0.0000 | 0.0005 | 0.0000 |
| f__Ranunculaceae;g__Isopyrum;s__*Isopyrum savilei* | 0.0000 | 0.0000 | 0.0000 | 0.0010 | 0.0000 | 0.0000 |
| f__Apiaceae;g__Chaerophyllum;s__*Chaerophyllum procumbens* | 0.0000 | 0.0000 | 0.0000 | 0.0000 | 0.0013 | 0.0014 |
| f__Brassicaceae;g__Draba;s__*Draba densifolia* | 0.0000 | 0.0000 | 0.0000 | 0.0000 | 0.0004 | 0.0005 |
| f__Caryophyllaceae;g__Cerastium;s__*Cerastium arvense* | 0.0000 | 0.0000 | 0.0000 | 0.0000 | 0.0000 | 0.0000 |
| f__Fabaceae;g__Craspedolobium;s__*Craspedolobium unijugum* | 0.0000 | 0.0000 | 0.0000 | 0.0000 | 0.0012 | 0.0004 |
| f__Caryophyllaceae;g__Stellaria;s__*Stellaria holostea* | 0.0000 | 0.0000 | 0.0000 | 0.0000 | 0.0000 | 0.0000 |
| f__Solanaceae;g__Solanum;s__*Solanum sp. Espinoza*5665 | 0.0000 | 0.0000 | 0.0000 | 0.0002 | 0.0004 | 0.0000 |
| f__Rosaceae;g__Prunus;s__*Prunus takesimensis* | 0.0000 | 0.0000 | 0.0000 | 0.0005 | 0.0000 | 0.0001 |
| f__Caryophyllaceae;g__Stellaria;s__*Stellaria americana* | 0.0000 | 0.0000 | 0.0000 | 0.0000 | 0.0000 | 0.0000 |
| f__Poaceae;g__Ctenium;s__*Ctenium floridanum* | 0.0000 | 0.0000 | 0.0000 | 0.0000 | 0.0012 | 0.0000 |
| f__Oxalidaceae;g__Oxalis;s__*Oxalis violacea* | 0.0000 | 0.0000 | 0.0000 | 0.0000 | 0.0000 | 0.0000 |
| f__Amaranthaceae;g__Achyranthes;s__*Achyranthes aspera* | 0.0000 | 0.0000 | 0.0000 | 0.0000 | 0.0008 | 0.0000 |
| f__Cucurbitaceae;g__Luffa;s__*Luffa quinquefida* | 0.0000 | 0.0000 | 0.0000 | 0.0000 | 0.0000 | 0.0000 |
| f__Rutaceae;g__Zanthoxylum;s__*Zanthoxylum clava-herculis* | 0.0000 | 0.0000 | 0.0000 | 0.0000 | 0.0000 | 0.0000 |
| f__Polygonaceae;g__Bistorta;s__*Bistorta officinalis* | 0.0000 | 0.0000 | 0.0000 | 0.0000 | 0.0000 | 0.0000 |
| f__Ginkgoaceae;g__Ginkgo;s__*Ginkgo biloba* | 0.0000 | 0.0000 | 0.0000 | 0.0000 | 0.0000 | 0.0000 |
| f__Poaceae;g__Sporobolus;s__*Sporobolus aculeatus* | 0.0000 | 0.0000 | 0.0000 | 0.0000 | 0.0000 | 0.0000 |
| f__Cornaceae;g__Cornus;s__*Cornus amomum* | 0.0000 | 0.0000 | 0.0000 | 0.0000 | 0.0000 | 0.0000 |
| f__Brassicaceae;g__Brassica;s__*Brassica rapa subsp. rapa* | 0.0000 | 0.0000 | 0.0000 | 0.0000 | 0.0002 | 0.0003 |
| f__Musaceae;g__Musa;s__*Musa hybrid cultivar* | 0.0000 | 0.0000 | 0.0000 | 0.0000 | 0.0000 | 0.0000 |
| f__Fabaceae;g__Pterocarpus;s__*Pterocarpus mildbraedii* | 0.0000 | 0.0000 | 0.0000 | 0.0000 | 0.0000 | 0.0000 |
| f__Cucurbitaceae;g__Corallocarpus;s__*Corallocarpus bainesii* | 0.0000 | 0.0000 | 0.0000 | 0.0003 | 0.0002 | 0.0002 |
| f__Caryophyllaceae;g__Cerastium;s__*Cerastium nutans* | 0.0000 | 0.0000 | 0.0000 | 0.0000 | 0.0000 | 0.0000 |
| f__Poaceae;g__Oryza;s__*Oryza meridionalis* | 0.0000 | 0.0000 | 0.0001 | 0.0000 | 0.0004 | 0.0010 |
| f__Rutaceae;g__Zanthoxylum;s__*Zanthoxylum simulans* | 0.0000 | 0.0000 | 0.0000 | 0.0000 | 0.0000 | 0.0000 |
| f__Juglandaceae;g__Cyclocarya;s__*Cyclocarya paliurus* | 0.0000 | 0.0000 | 0.0000 | 0.0000 | 0.0000 | 0.0000 |
| f__Oxalidaceae;g__Oxalis;s__*Oxalis oregana* | 0.0000 | 0.0000 | 0.0000 | 0.0000 | 0.0000 | 0.0000 |
| f__Lauraceae;g__Cryptocarya;s__*Cryptocarya bidwillii* | 0.0000 | 0.0000 | 0.0000 | 0.0000 | 0.0002 | 0.0006 |
| f__Corynocarpaceae;g__Corynocarpus;s__*Corynocarpus rupestris* | 0.0000 | 0.0000 | 0.0000 | 0.0000 | 0.0000 | 0.0000 |
| f__Rutaceae;g__Zanthoxylum;s__*Zanthoxylum americanum* | 0.0000 | 0.0000 | 0.0000 | 0.0000 | 0.0000 | 0.0000 |
| f__Musaceae;g__Ensete;s__*Ensete ventricosum* | 0.0000 | 0.0000 | 0.0000 | 0.0000 | 0.0000 | 0.0000 |
| f__Polygonaceae;g__Bistorta;s__*Bistorta bistortoides* | 0.0000 | 0.0000 | 0.0000 | 0.0000 | 0.0000 | 0.0000 |
| f__Solanaceae;g__Solanum;s__*Solanum giganteum* | 0.0001 | 0.0000 | 0.0000 | 0.0001 | 0.0000 | 0.0000 |
| f__Cornaceae;g__Cornus;s__*Cornus racemosa* | 0.0000 | 0.0000 | 0.0000 | 0.0000 | 0.0000 | 0.0000 |
| f__Euphorbiaceae;g__Acalypha;s__*Acalypha rhomboidea* | 0.0000 | 0.0001 | 0.0001 | 0.0000 | 0.0000 | 0.0001 |
| f__Fabaceae;g__Pterocarpus;s__*Pterocarpus soyauxii* | 0.0000 | 0.0000 | 0.0000 | 0.0000 | 0.0000 | 0.0000 |
| f__Cucurbitaceae;g__Cucumis;s__*Cucumis melo* | 0.0000 | 0.0000 | 0.0000 | 0.0000 | 0.0000 | 0.0007 |
| f__Pylaisiadelphaceae;g__Brotherella;s__*Brotherella recurvans* | 0.0000 | 0.0000 | 0.0000 | 0.0000 | 0.0000 | 0.0000 |
| f__Cucurbitaceae;g__Thladiantha;s__*Thladiantha villosula* | 0.0000 | 0.0000 | 0.0000 | 0.0000 | 0.0000 | 0.0000 |
| f__Corynocarpaceae;g__Corynocarpus;s__*Corynocarpus laevigatus* | 0.0000 | 0.0000 | 0.0000 | 0.0000 | 0.0005 | 0.0000 |
| f__Lauraceae;g__Cinnamomum;s__*Cinnamomum aromaticum* | 0.0000 | 0.0000 | 0.0000 | 0.0000 | 0.0000 | 0.0000 |
| f__Rosaceae;g__Prunus;s__*Prunus longistyla* | 0.0000 | 0.0000 | 0.0000 | 0.0000 | 0.0000 | 0.0000 |
| f__Fabaceae;g__Medicago;s__*Medicago rotata* | 0.0000 | 0.0000 | 0.0000 | 0.0000 | 0.0000 | 0.0000 |
| f__Caryophyllaceae;g__Cerastium;s__*Cerastium tianschanicum* | 0.0000 | 0.0000 | 0.0000 | 0.0000 | 0.0000 | 0.0000 |
| f__Euphorbiaceae;g__Cnidoscolus;s__*Cnidoscolus urens* | 0.0000 | 0.0000 | 0.0001 | 0.0000 | 0.0000 | 0.0000 |
| f__Cucurbitaceae;g__Melothria;s__*Melothria trilobata* | 0.0000 | 0.0000 | 0.0000 | 0.0000 | 0.0001 | 0.0001 |
| f__Poaceae;g__Oryza;s__*Oryza ridleyi* | 0.0000 | 0.0000 | 0.0000 | 0.0000 | 0.0000 | 0.0000 |
| f__Solanaceae;g__Solanum;s__*Solanum tuberosum* | 0.0000 | 0.0000 | 0.0003 | 0.0000 | 0.0000 | 0.0000 |
| Supplementary Table 2.4 Relative abundance of plant food items at the species level in all Chinese mole shrew diet samples during winter. | | | | | | |
| Taxon | Relative abundance (Winter) | | | | | |
|  | W1286a | W1286b | W1286c | W1287a | W1287b | W1287c |
| f__Fabaceae;g__Arachis;s__*Arachis hypogaea* | 0.4198 | 0.0000 | 0.1522 | 0.8471 | 0.8480 | 0.8681 |
| f__Poaceae;g__Chikusichloa;s__*Chikusichloa aquatica* | 0.1400 | 0.1130 | 0.0744 | 0.0001 | 0.0001 | 0.0000 |
| f__Lauraceae;g__Cinnamomum;s__*Cinnamomum glaucescens* | 0.0017 | 0.0004 | 0.0112 | 0.0015 | 0.0018 | 0.0006 |
| f__Caryophyllaceae;g__Cerastium;s__*Cerastium glomeratum* | 0.0001 | 0.0000 | 0.0002 | 0.1185 | 0.1187 | 0.0961 |
| f__Oleaceae;g__Nestegis;s__*Nestegis apetala* | 0.0000 | 0.0418 | 0.0000 | 0.0009 | 0.0002 | 0.0002 |
| f__unidentified;g__Camptotheca;s__*Camptotheca acuminata* | 0.0024 | 0.0016 | 0.0000 | 0.0000 | 0.0001 | 0.0001 |
| f__Caryophyllaceae;g__Stellaria;s__*Stellaria media* | 0.0000 | 0.0000 | 0.0000 | 0.0018 | 0.0017 | 0.0047 |
| f__Phytolaccaceae;g__Phytolacca;s__*Phytolacca americana* | 0.0000 | 0.0000 | 0.0000 | 0.0000 | 0.0000 | 0.0000 |
| f__Poaceae;g__Oryza;s__*Oryza sativa* | 0.0839 | 0.0167 | 0.0003 | 0.0001 | 0.0003 | 0.0001 |
| f__Cyperaceae;g__Carex;s__*Carex aneurocarpa* | 0.0000 | 0.0018 | 0.0000 | 0.0000 | 0.0000 | 0.0000 |
| f__Iridaceae;g__Gladiolus;s__*Gladiolus x gandavensis* | 0.0000 | 0.4804 | 0.0000 | 0.0000 | 0.0000 | 0.0000 |
| f__Solanaceae;g__Withania;s__*Withania frutescen*s | 0.0000 | 0.0000 | 0.0000 | 0.0000 | 0.0001 | 0.0000 |
| f__Musaceae;g__Musa;s__*Musa laterita* | 0.1895 | 0.0281 | 0.0000 | 0.0000 | 0.0000 | 0.0000 |
| f__Lauraceae;g__Cinnamomum;s__*Cinnamomum bodinieri* | 0.1309 | 0.0862 | 0.2526 | 0.0001 | 0.0002 | 0.0000 |
| f__Mazaceae;g__Mazus;s__*Mazus reptans* | 0.0001 | 0.0000 | 0.0000 | 0.0000 | 0.0000 | 0.0000 |
| f__Asteraceae;g__Lactuca;s__*Lactuca sativa* | 0.0000 | 0.0000 | 0.3685 | 0.0001 | 0.0000 | 0.0001 |
| f__Asteraceae;g__Galinsoga;s__*Galinsoga parviflora* | 0.0000 | 0.0000 | 0.0003 | 0.0000 | 0.0000 | 0.0000 |
| f__Ranunculaceae;g__Isopyrum;s__*Isopyrum biternatum* | 0.0000 | 0.0000 | 0.0000 | 0.0000 | 0.0000 | 0.0000 |
| f__Rosaceae;g__Prunus;s__*Prunus mongolica* | 0.0000 | 0.0000 | 0.0000 | 0.0000 | 0.0000 | 0.0001 |
| f__Pedaliaceae;g__Uncarina;s__*Uncarina grandidieri* | 0.0000 | 0.0001 | 0.0000 | 0.0000 | 0.0000 | 0.0000 |
| f__Fabaceae;g__Cyamopsis;s__*Cyamopsis tetragonoloba* | 0.0000 | 0.0000 | 0.0000 | 0.0000 | 0.0000 | 0.0000 |
| f__Linderniaceae;g__Lindernia;s__*Lindernia antipoda* | 0.0000 | 0.0000 | 0.0000 | 0.0000 | 0.0000 | 0.0000 |
| f__Asteraceae;g__Leontopodium;s__*Leontopodium alpinum* | 0.0000 | 0.0000 | 0.0061 | 0.0000 | 0.0000 | 0.0000 |
| f__Poaceae;g__Eleusine;s__*Eleusine indica* | 0.0000 | 0.0001 | 0.0000 | 0.0000 | 0.0000 | 0.0000 |
| f__Polygonaceae;g__Bistorta;s__*Bistorta vivipara* | 0.0000 | 0.0000 | 0.0000 | 0.0000 | 0.0000 | 0.0000 |
| f__Juglandaceae;g__Carya;s__*Carya glabra* | 0.0000 | 0.0000 | 0.0000 | 0.0000 | 0.0000 | 0.0001 |
| f__Poaceae;g__Orinus;s__*Orinus kokonoricus* | 0.0000 | 0.0000 | 0.0000 | 0.0000 | 0.0000 | 0.0000 |
| f__Fabaceae;g__Medicago;s__*Medicago sativa* | 0.0000 | 0.0004 | 0.0000 | 0.0000 | 0.0000 | 0.0000 |
| f__Alismataceae;g__Sagittaria;s__*Sagittaria trifolia* | 0.0000 | 0.0000 | 0.0000 | 0.0000 | 0.0000 | 0.0000 |
| f__Amaryllidaceae;g__Allium;s__*Allium stellatum* | 0.0000 | 0.0033 | 0.1220 | 0.0000 | 0.0000 | 0.0000 |
| f__Euphorbiaceae;g__Euphorbia;s__*Euphorbia hypericifolia* | 0.0000 | 0.0976 | 0.0000 | 0.0000 | 0.0000 | 0.0000 |
| f__Amaryllidaceae;g__Allium;s__*Allium prattii* | 0.0000 | 0.0443 | 0.0078 | 0.0000 | 0.0000 | 0.0000 |
| f__Euphorbiaceae;g__Acalypha;s__*Acalypha australis* | 0.0263 | 0.0057 | 0.0000 | 0.0000 | 0.0000 | 0.0000 |
| f__Asteraceae;g__Faberia;s__*Faberia pinnatifida* | 0.0000 | 0.0000 | 0.0007 | 0.0000 | 0.0000 | 0.0000 |
| f__Pylaisiadelphaceae;g__Pylaisiadelpha;s__*Pylaisiadelpha tenuirostris* | 0.0000 | 0.0000 | 0.0000 | 0.0000 | 0.0000 | 0.0000 |
| f__Apiaceae;g__Ligusticum;s__*Ligusticum sinense* | 0.0000 | 0.0000 | 0.0000 | 0.0006 | 0.0006 | 0.0001 |
| f__Rubiaceae;g__Uncaria;s__*Uncaria tomentosa* | 0.0000 | 0.0000 | 0.0000 | 0.0000 | 0.0000 | 0.0000 |
| f__Hypnaceae;g__Callicladium;s__*Callicladium haldanianum* | 0.0000 | 0.0000 | 0.0000 | 0.0000 | 0.0000 | 0.0000 |
| f__Fabaceae;g__Medicago;s__*Medicago laciniata* | 0.0000 | 0.0599 | 0.0000 | 0.0000 | 0.0000 | 0.0000 |
| f__Rosaceae;g__Rosa;s__*Rosa chinensis var. spontanea* | 0.0000 | 0.0000 | 0.0000 | 0.0000 | 0.0000 | 0.0000 |
| f__Rubiaceae;g__Ophiorrhiza;s__*Ophiorrhiza sp. SH-2010* | 0.0000 | 0.0003 | 0.0000 | 0.0000 | 0.0000 | 0.0000 |
| f__Oleaceae;g__Osmanthus;s__*Osmanthus americanus* | 0.0000 | 0.0018 | 0.0000 | 0.0000 | 0.0000 | 0.0000 |
| f__Polygonaceae;g__Rumex;s__*Rumex utahensis* | 0.0000 | 0.0000 | 0.0000 | 0.0000 | 0.0000 | 0.0000 |
| f__Musaceae;g__Musa;s__*Musa acuminata* | 0.0021 | 0.0016 | 0.0000 | 0.0000 | 0.0000 | 0.0000 |
| f__Moraceae;g__Ficus;s__*Ficus benghalensis* | 0.0000 | 0.0000 | 0.0000 | 0.0000 | 0.0000 | 0.0000 |
| f__Cucurbitaceae;g__Cucurbita;s__*Cucurbita pepo* | 0.0000 | 0.0000 | 0.0000 | 0.0002 | 0.0006 | 0.0002 |
| f__Convolvulaceae;g__Convolvulus;s__*Convolvulus arvensis* | 0.0000 | 0.0000 | 0.0000 | 0.0000 | 0.0000 | 0.0000 |
| f__Poaceae;g__Oryza;s__*Oryza rhizomatis* | 0.0007 | 0.0001 | 0.0000 | 0.0000 | 0.0000 | 0.0000 |
| f__Brassicaceae;g__Brassica;s__*Brassica oleracea var. botrytis* | 0.0000 | 0.0000 | 0.0000 | 0.0018 | 0.0012 | 0.0014 |
| f__Asteraceae;g__Helichrysum;s__*Helichrysum zeyheri* | 0.0000 | 0.0000 | 0.0016 | 0.0000 | 0.0000 | 0.0000 |
| f__Araceae;g__Lemna;s__*Lemna minor* | 0.0000 | 0.0000 | 0.0000 | 0.0000 | 0.0000 | 0.0000 |
| f__Cornaceae;g__Cornus;s__*Cornus sericea* | 0.0000 | 0.0000 | 0.0000 | 0.0000 | 0.0000 | 0.0000 |
| f__Betulaceae;g__Alnus;s__*Alnus rubra* | 0.0000 | 0.0000 | 0.0000 | 0.0000 | 0.0000 | 0.0000 |
| f__Solanaceae;g__Capsicum;s__*Capsicum annuum* | 0.0000 | 0.0000 | 0.0000 | 0.0000 | 0.0000 | 0.0000 |
| f__Iridaceae;g__Gladiolus;s__*Gladiolus palustris* | 0.0000 | 0.0131 | 0.0000 | 0.0000 | 0.0000 | 0.0000 |
| f__Cucurbitaceae;g__Cucumis;s__*Cucumis sativus* | 0.0000 | 0.0000 | 0.0000 | 0.0004 | 0.0003 | 0.0006 |
| f__Cucurbitaceae;g__Cucurbita;s__*Cucurbita moschata* | 0.0000 | 0.0000 | 0.0000 | 0.0000 | 0.0000 | 0.0000 |
| f__Fabaceae;g__Glycine;s__*Glycine canescens* | 0.0000 | 0.0000 | 0.0000 | 0.0000 | 0.0000 | 0.0000 |
| f__Oxalidaceae;g__Oxalis;s__*Oxalis debilis* | 0.0000 | 0.0000 | 0.0000 | 0.0000 | 0.0000 | 0.0000 |
| f__Fabaceae;g__Kummerowia;s__*Kummerowia striata* | 0.0000 | 0.0000 | 0.0000 | 0.0050 | 0.0054 | 0.0055 |
| f__Fabaceae;g__Stylosanthes;s__*Stylosanthes viscosa* | 0.0007 | 0.0000 | 0.0018 | 0.0047 | 0.0030 | 0.0049 |
| f__Achatocarpaceae;g__Achatocarpus;s__*Achatocarpus gracilis* | 0.0000 | 0.0000 | 0.0000 | 0.0001 | 0.0001 | 0.0000 |
| f__Poaceae;g__Oryza;s__*Oryza longiglumis* | 0.0002 | 0.0006 | 0.0000 | 0.0000 | 0.0000 | 0.0000 |
| f__Fabaceae;g__Pterocarpus;s__*Pterocarpus ternatus* | 0.0000 | 0.0000 | 0.0000 | 0.0007 | 0.0006 | 0.0007 |
| f__Solanaceae;g__Lycium;s__*Lycium chinense* | 0.0000 | 0.0000 | 0.0000 | 0.0000 | 0.0000 | 0.0000 |
| f__Ranunculaceae;g__Isopyrum;s__*Isopyrum savilei* | 0.0000 | 0.0000 | 0.0000 | 0.0000 | 0.0000 | 0.0000 |
| f__Apiaceae;g__Chaerophyllum;s__*Chaerophyllum procumbens* | 0.0000 | 0.0000 | 0.0000 | 0.0026 | 0.0033 | 0.0020 |
| f__Brassicaceae;g__Draba;s__*Draba densifolia* | 0.0000 | 0.0000 | 0.0000 | 0.0030 | 0.0023 | 0.0028 |
| f__Caryophyllaceae;g__Cerastium;s__*Cerastium arvense* | 0.0000 | 0.0000 | 0.0000 | 0.0018 | 0.0022 | 0.0019 |
| f__Fabaceae;g__Craspedolobium;s__*Craspedolobium unijugum* | 0.0000 | 0.0000 | 0.0000 | 0.0005 | 0.0006 | 0.0006 |
| f__Caryophyllaceae;g__Stellaria;s__*Stellaria holostea* | 0.0000 | 0.0000 | 0.0000 | 0.0026 | 0.0027 | 0.0025 |
| f__Solanaceae;g__Solanum;s__*Solanum sp. Espinoza*5665 | 0.0000 | 0.0000 | 0.0000 | 0.0000 | 0.0001 | 0.0000 |
| f__Rosaceae;g__Prunus;s__*Prunus takesimensis* | 0.0000 | 0.0000 | 0.0000 | 0.0001 | 0.0000 | 0.0001 |
| f__Caryophyllaceae;g__Stellaria;s__*Stellaria americana* | 0.0000 | 0.0000 | 0.0000 | 0.0001 | 0.0001 | 0.0000 |
| f__Poaceae;g__Ctenium;s__*Ctenium floridanum* | 0.0000 | 0.0000 | 0.0000 | 0.0000 | 0.0000 | 0.0000 |
| f__Oxalidaceae;g__Oxalis;s__*Oxalis violacea* | 0.0000 | 0.0000 | 0.0000 | 0.0000 | 0.0000 | 0.0000 |
| f__Amaranthaceae;g__Achyranthes;s__*Achyranthes aspera* | 0.0000 | 0.0000 | 0.0000 | 0.0000 | 0.0000 | 0.0000 |
| f__Cucurbitaceae;g__Luffa;s__*Luffa quinquefida* | 0.0000 | 0.0000 | 0.0000 | 0.0000 | 0.0000 | 0.0000 |
| f__Rutaceae;g__Zanthoxylum;s__*Zanthoxylum clava-herculis* | 0.0000 | 0.0000 | 0.0000 | 0.0015 | 0.0017 | 0.0014 |
| f__Polygonaceae;g__Bistorta;s__*Bistorta officinalis* | 0.0000 | 0.0000 | 0.0000 | 0.0000 | 0.0000 | 0.0000 |
| f__Ginkgoaceae;g__Ginkgo;s__*Ginkgo biloba* | 0.0000 | 0.0000 | 0.0000 | 0.0000 | 0.0000 | 0.0000 |
| f__Poaceae;g__Sporobolus;s__*Sporobolus aculeatus* | 0.0000 | 0.0000 | 0.0000 | 0.0000 | 0.0000 | 0.0000 |
| f__Cornaceae;g__Cornus;s__*Cornus amomum* | 0.0000 | 0.0000 | 0.0000 | 0.0000 | 0.0000 | 0.0000 |
| f__Brassicaceae;g__Brassica;s__*Brassica rapa subsp. rapa* | 0.0000 | 0.0000 | 0.0000 | 0.0005 | 0.0003 | 0.0003 |
| f__Musaceae;g__Musa;s__*Musa hybrid cultivar* | 0.0010 | 0.0001 | 0.0000 | 0.0000 | 0.0000 | 0.0000 |
| f__Fabaceae;g__Pterocarpus;s__*Pterocarpus mildbraedii* | 0.0000 | 0.0000 | 0.0000 | 0.0008 | 0.0008 | 0.0011 |
| f__Cucurbitaceae;g__Corallocarpus;s__*Corallocarpus bainesii* | 0.0000 | 0.0000 | 0.0000 | 0.0005 | 0.0007 | 0.0008 |
| f__Caryophyllaceae;g__Cerastium;s__*Cerastium nutans* | 0.0000 | 0.0000 | 0.0000 | 0.0004 | 0.0003 | 0.0004 |
| f__Poaceae;g__Oryza;s__*Oryza meridionalis* | 0.0000 | 0.0000 | 0.0001 | 0.0000 | 0.0000 | 0.0000 |
| f__Rutaceae;g__Zanthoxylum;s__*Zanthoxylum simulans* | 0.0000 | 0.0000 | 0.0000 | 0.0006 | 0.0007 | 0.0007 |
| f__Juglandaceae;g__Cyclocarya;s__*Cyclocarya paliurus* | 0.0000 | 0.0000 | 0.0000 | 0.0000 | 0.0000 | 0.0000 |
| f__Oxalidaceae;g__Oxalis;s__*Oxalis oregana* | 0.0000 | 0.0000 | 0.0000 | 0.0000 | 0.0000 | 0.0000 |
| f__Lauraceae;g__Cryptocarya;s__*Cryptocarya bidwillii* | 0.0000 | 0.0000 | 0.0000 | 0.0000 | 0.0000 | 0.0000 |
| f__Corynocarpaceae;g__Corynocarpus;s__*Corynocarpus rupestris* | 0.0000 | 0.0000 | 0.0001 | 0.0003 | 0.0003 | 0.0004 |
| f__Rutaceae;g__Zanthoxylum;s__*Zanthoxylum americanum* | 0.0000 | 0.0000 | 0.0000 | 0.0004 | 0.0003 | 0.0005 |
| f__Musaceae;g__Ensete;s__*Ensete ventricosum* | 0.0000 | 0.0000 | 0.0000 | 0.0000 | 0.0000 | 0.0000 |
| f__Polygonaceae;g__Bistorta;s__*Bistorta bistortoides* | 0.0000 | 0.0000 | 0.0000 | 0.0000 | 0.0000 | 0.0000 |
| f__Solanaceae;g__Solanum;s__*Solanum giganteum* | 0.0000 | 0.0000 | 0.0000 | 0.0000 | 0.0000 | 0.0000 |
| f__Cornaceae;g__Cornus;s__*Cornus racemosa* | 0.0000 | 0.0000 | 0.0000 | 0.0000 | 0.0000 | 0.0000 |
| f__Euphorbiaceae;g__Acalypha;s__*Acalypha rhomboidea* | 0.0004 | 0.0002 | 0.0000 | 0.0000 | 0.0000 | 0.0000 |
| f__Fabaceae;g__Pterocarpus;s__*Pterocarpus soyauxii* | 0.0000 | 0.0000 | 0.0000 | 0.0003 | 0.0001 | 0.0003 |
| f__Cucurbitaceae;g__Cucumis;s__*Cucumis melo* | 0.0000 | 0.0000 | 0.0000 | 0.0000 | 0.0000 | 0.0000 |
| f__Pylaisiadelphaceae;g__Brotherella;s__*Brotherella recurvans* | 0.0000 | 0.0000 | 0.0000 | 0.0000 | 0.0000 | 0.0000 |
| f__Cucurbitaceae;g__Thladiantha;s__*Thladiantha villosula* | 0.0000 | 0.0000 | 0.0000 | 0.0001 | 0.0001 | 0.0002 |
| f__Corynocarpaceae;g__Corynocarpus;s__*Corynocarpus laevigatus* | 0.0000 | 0.0000 | 0.0000 | 0.0000 | 0.0000 | 0.0000 |
| f__Lauraceae;g__Cinnamomum;s__*Cinnamomum aromaticum* | 0.0000 | 0.0000 | 0.0000 | 0.0000 | 0.0000 | 0.0000 |
| f__Rosaceae;g__Prunus;s__*Prunus longistyla* | 0.0000 | 0.0000 | 0.0000 | 0.0000 | 0.0000 | 0.0000 |
| f__Fabaceae;g__Medicago;s__*Medicago rotata* | 0.0000 | 0.0004 | 0.0000 | 0.0000 | 0.0000 | 0.0000 |
| f__Caryophyllaceae;g__Cerastium;s__*Cerastium tianschanicum* | 0.0000 | 0.0000 | 0.0000 | 0.0001 | 0.0002 | 0.0000 |
| f__Euphorbiaceae;g__Cnidoscolus;s__*Cnidoscolus urens* | 0.0001 | 0.0000 | 0.0000 | 0.0000 | 0.0000 | 0.0000 |
| f__Cucurbitaceae;g__Melothria;s__*Melothria trilobata* | 0.0000 | 0.0000 | 0.0000 | 0.0000 | 0.0000 | 0.0000 |
| f__Poaceae;g__Oryza;s__*Oryza ridleyi* | 0.0000 | 0.0001 | 0.0000 | 0.0000 | 0.0000 | 0.0000 |
| f__Solanaceae;g__Solanum;s__*Solanum tuberosum* | 0.0000 | 0.0000 | 0.0000 | 0.0000 | 0.0000 | 0.0000 |
