# Supplementary material for: DNA metabarcoding provides insights into seasonal diet variations in Chinese mole shrew (Anourosorex squamipes) with potential implications for evaluating crop impacts
Source: Ecol Evol. 2020 Nov 25;11(1):376–89. doi: 10.1002/ece3.7055 (PMC7790647; doi:10.1002/ece3.7055)
Supplement: Supplementary file 3 — Table S3 [file ECE3-11-376-s003.doc]

Supplementary Table 3. Relative abundance of animal food items at the order level in all Chinese mole shrew diet samples.

| Order-level | Relative abundance (Spring) | | | | | | Relative abundance (Summer) | | | | | |
| --- | --- | --- | --- | --- | --- | --- | --- | --- | --- | --- | --- | --- |
|  | Sp1306a | Sp1306b | Sp1306c | Sp1309a | Sp1309b | Sp1309c | Su1560a | Su1560b | Su1560c | Su1568a | Su1568b | Su1568c |
| Haplotaxida | 0.9387 | 0.9042 | 0.9221 | 0.6906 | 0.7583 | 0.7626 | 0.7190 | 0.5092 | 0.6868 | 0.9980 | 0.9856 | 0.9892 |
| Stylommatophora | 0.0224 | 0.0335 | 0.0312 | 0.0000 | 0.0000 | 0.0009 | 0.0065 | 0.0086 | 0.0061 | 0.0010 | 0.0011 | 0.0019 |
| Hymenoptera | 0.0000 | 0.0000 | 0.0000 | 0.0000 | 0.0000 | 0.0000 | 0.0000 | 0.0000 | 0.0000 | 0.0000 | 0.0000 | 0.0000 |
| Orthoptera | 0.0026 | 0.0044 | 0.0015 | 0.0630 | 0.0525 | 0.0555 | 0.2579 | 0.4597 | 0.2832 | 0.0007 | 0.0018 | 0.0003 |
| Moniligastrida | 0.0339 | 0.0514 | 0.0437 | 0.2365 | 0.1786 | 0.1733 | 0.0003 | 0.0003 | 0.0005 | 0.0000 | 0.0000 | 0.0000 |
| Araneae | 0.0000 | 0.0000 | 0.0000 | 0.0000 | 0.0000 | 0.0000 | 0.0000 | 0.0000 | 0.0019 | 0.0000 | 0.0000 | 0.0000 |
| Coleoptera | 0.0000 | 0.0000 | 0.0000 | 0.0000 | 0.0000 | 0.0000 | 0.0000 | 0.0000 | 0.0000 | 0.0000 | 0.0085 | 0.0066 |
| Dermaptera | 0.0000 | 0.0000 | 0.0000 | 0.0000 | 0.0000 | 0.0000 | 0.0127 | 0.0218 | 0.0146 | 0.0000 | 0.0000 | 0.0000 |
| Lithobiomorpha | 0.0000 | 0.0000 | 0.0000 | 0.0089 | 0.0101 | 0.0077 | 0.0000 | 0.0000 | 0.0000 | 0.0000 | 0.0000 | 0.0000 |
| Diptera | 0.0020 | 0.0055 | 0.0011 | 0.0000 | 0.0000 | 0.0000 | 0.0000 | 0.0000 | 0.0000 | 0.0000 | 0.0000 | 0.0000 |
| Lepidoptera | 0.0000 | 0.0000 | 0.0000 | 0.0000 | 0.0000 | 0.0000 | 0.0000 | 0.0000 | 0.0000 | 0.0000 | 0.0000 | 0.0013 |
| Rodentia | 0.0003 | 0.0000 | 0.0000 | 0.0000 | 0.0000 | 0.0000 | 0.0012 | 0.0000 | 0.0000 | 0.0000 | 0.0000 | 0.0000 |
| Blattodea | 0.0000 | 0.0000 | 0.0000 | 0.0000 | 0.0000 | 0.0000 | 0.0015 | 0.0000 | 0.0066 | 0.0000 | 0.0000 | 0.0000 |
| Amphipoda | 0.0000 | 0.0011 | 0.0004 | 0.0009 | 0.0005 | 0.0000 | 0.0009 | 0.0003 | 0.0003 | 0.0003 | 0.0000 | 0.0006 |
| Hemiptera | 0.0000 | 0.0000 | 0.0000 | 0.0000 | 0.0000 | 0.0000 | 0.0000 | 0.0000 | 0.0000 | 0.0000 | 0.0029 | 0.0000 |

| Order-level | Relative abundance (Autumn) | | | | | | Relative abundance (Winter) | | | | | |
| --- | --- | --- | --- | --- | --- | --- | --- | --- | --- | --- | --- | --- |
|  | A1003a | A1003b | A1003c | A1011a | A1011b | A1011c | W1286a | W1286b | W1286c | W1287a | W1287b | W1287c |
| Haplotaxida | 0.5365 | 0.9808 | 0.9724 | 0.9458 | 0.9943 | 0.9916 | 0.5833 | 0.2414 | 0.3478 | 0.4400 | 0.5500 | 0.5333 |
| Stylommatophora | 0.0000 | 0.0000 | 0.0000 | 0.0000 | 0.0000 | 0.0000 | 0.4167 | 0.7586 | 0.6522 | 0.0000 | 0.0000 | 0.0000 |
| Hymenoptera | 0.0000 | 0.0000 | 0.0000 | 0.0000 | 0.0000 | 0.0000 | 0.0000 | 0.0000 | 0.0000 | 0.5600 | 0.4500 | 0.4667 |
| Orthoptera | 0.0584 | 0.0137 | 0.0214 | 0.0000 | 0.0000 | 0.0000 | 0.0000 | 0.0000 | 0.0000 | 0.0000 | 0.0000 | 0.0000 |
| Moniligastrida | 0.0000 | 0.0000 | 0.0000 | 0.0404 | 0.0022 | 0.0031 | 0.0000 | 0.0000 | 0.0000 | 0.0000 | 0.0000 | 0.0000 |
| Araneae | 0.3504 | 0.0000 | 0.0000 | 0.0000 | 0.0000 | 0.0000 | 0.0000 | 0.0000 | 0.0000 | 0.0000 | 0.0000 | 0.0000 |
| Coleoptera | 0.0474 | 0.0023 | 0.0014 | 0.0000 | 0.0000 | 0.0000 | 0.0000 | 0.0000 | 0.0000 | 0.0000 | 0.0000 | 0.0000 |
| Dermaptera | 0.0000 | 0.0000 | 0.0000 | 0.0000 | 0.0000 | 0.0000 | 0.0000 | 0.0000 | 0.0000 | 0.0000 | 0.0000 | 0.0000 |
| Lithobiomorpha | 0.0000 | 0.0000 | 0.0000 | 0.0000 | 0.0000 | 0.0000 | 0.0000 | 0.0000 | 0.0000 | 0.0000 | 0.0000 | 0.0000 |
| Diptera | 0.0073 | 0.0000 | 0.0000 | 0.0023 | 0.0008 | 0.0017 | 0.0000 | 0.0000 | 0.0000 | 0.0000 | 0.0000 | 0.0000 |
| Lepidoptera | 0.0000 | 0.0000 | 0.0000 | 0.0115 | 0.0011 | 0.0031 | 0.0000 | 0.0000 | 0.0000 | 0.0000 | 0.0000 | 0.0000 |
| Rodentia | 0.0000 | 0.0031 | 0.0048 | 0.0000 | 0.0016 | 0.0006 | 0.0000 | 0.0000 | 0.0000 | 0.0000 | 0.0000 | 0.0000 |
| Blattodea | 0.0000 | 0.0000 | 0.0000 | 0.0000 | 0.0000 | 0.0000 | 0.0000 | 0.0000 | 0.0000 | 0.0000 | 0.0000 | 0.0000 |
| Amphipoda | 0.0000 | 0.0000 | 0.0000 | 0.0000 | 0.0000 | 0.0000 | 0.0000 | 0.0000 | 0.0000 | 0.0000 | 0.0000 | 0.0000 |
| Hemiptera | 0.0000 | 0.0000 | 0.0000 | 0.0000 | 0.0000 | 0.0000 | 0.0000 | 0.0000 | 0.0000 | 0.0000 | 0.0000 | 0.0000 |
