# Supplementary material for: DNA metabarcoding provides insights into seasonal diet variations in Chinese mole shrew (Anourosorex squamipes) with potential implications for evaluating crop impacts
Source: Ecol Evol. 2020 Nov 25;11(1):376–89. doi: 10.1002/ece3.7055 (PMC7790647; doi:10.1002/ece3.7055)
Supplement: Supplementary file 4 — Table S4 [file ECE3-11-376-s004.doc]

Supplementary Table 4. FO (frequency of occurrence) and relative abundance of earthworms at the species level in all Chinese mole shrew diet samples throughout the year.

| Taxon | Spring | | | | | | | Summer | | | | | | |
| --- | --- | --- | --- | --- | --- | --- | --- | --- | --- | --- | --- | --- | --- | --- |
| Species Level | Relative abundance of different individuals | | | | | | FO | Relative abundance of different individuals | | | | | | FO |
|  | Sp1306a | Sp1306b | Sp1306c | Sp1309a | Sp1309b | Sp1309c |  | Su1560a | Su1560b | Su1560c | Su1568a | Su1568b | Su1568c |  |
| *Metaphire californica* | 0.541 | 0.579 | 0.572 | 0.007 | 0.003 | 0.003 | 100% | 0.010 | 0.010 | 0.009 | 0.357 | 0.397 | 0.403 | 100% |
| *Amynthas morrisi* | 0.000 | 0.004 | 0.003 | 0.637 | 0.695 | 0.704 | 83% | 0.065 | 0.152 | 0.115 | 0.602 | 0.580 | 0.560 | 100% |
| *Amynthas corticis* | 0.056 | 0.070 | 0.067 | 0.046 | 0.058 | 0.054 | 100% | 0.553 | 0.228 | 0.424 | 0.012 | 0.003 | 0.019 | 100% |
| *Enchytraeus japonensis* | 0.306 | 0.225 | 0.247 | 0.001 | 0.001 | 0.001 | 100% | 0.001 | 0.000 | 0.000 | 0.000 | 0.000 | 0.001 | 67% |
| *Drawida sp. Watarase* | 0.000 | 0.000 | 0.000 | 0.235 | 0.174 | 0.173 | 67% | 0.000 | 0.000 | 0.001 | 0.000 | 0.000 | 0.000 | 33% |
| *Aporrectodea aff.* | 0.002 | 0.001 | 0.005 | 0.001 | 0.001 | 0.001 | 100% | 0.017 | 0.015 | 0.011 | 0.018 | 0.005 | 0.005 | 100% |
| *Amynthas gracilis* | 0.000 | 0.000 | 0.000 | 0.000 | 0.000 | 0.000 | 17% | 0.063 | 0.093 | 0.118 | 0.000 | 0.000 | 0.000 | 67% |
| *Drawida koreana* | 0.034 | 0.051 | 0.044 | 0.000 | 0.000 | 0.000 | 67% | 0.000 | 0.000 | 0.000 | 0.000 | 0.000 | 0.000 | 17% |
| *Bimastos palustris* | 0.020 | 0.017 | 0.018 | 0.000 | 0.000 | 0.000 | 100% | 0.010 | 0.011 | 0.008 | 0.000 | 0.000 | 0.000 | 50% |
| *Henlea perpusilla* | 0.009 | 0.005 | 0.005 | 0.000 | 0.000 | 0.000 | 50% | 0.000 | 0.000 | 0.000 | 0.000 | 0.000 | 0.000 | 0% |
| *Amynthas hupeiensis* | 0.004 | 0.004 | 0.004 | 0.000 | 0.000 | 0.000 | 50% | 0.000 | 0.000 | 0.000 | 0.000 | 0.000 | 0.000 | 0% |
| *Megascolecidae sp.* | 0.000 | 0.000 | 0.000 | 0.000 | 0.000 | 0.000 | 0% | 0.001 | 0.001 | 0.001 | 0.000 | 0.000 | 0.000 | 50% |

| Taxon | Autumn | | | | | | | Winter | | | | | | | Yearly |
| --- | --- | --- | --- | --- | --- | --- | --- | --- | --- | --- | --- | --- | --- | --- | --- |
| Species Level | Relative abundance of different individuals | | | | | | FO | Relative abundance of different individuals | | | | | | FO | FO |
|  | A1003a | A1003b | A1003c | A1011a | A1011b | A1011c |  | W1286a | W1286b | W1286c | W1287a | W1287b | W1287c |  |  |
| *Metaphire californica* | 0.518 | 0.974 | 0.966 | 0.264 | 0.424 | 0.453 | 100% | 0.375 | 0.138 | 0.217 | 0.280 | 0.250 | 0.467 | 100% | 100% |
| *Amynthas morrisi* | 0.000 | 0.001 | 0.000 | 0.051 | 0.067 | 0.067 | 67% | 0.000 | 0.000 | 0.087 | 0.160 | 0.050 | 0.000 | 50% | 75% |
| *Amynthas corticis* | 0.007 | 0.005 | 0.006 | 0.595 | 0.491 | 0.459 | 100% | 0.208 | 0.103 | 0.000 | 0.000 | 0.150 | 0.000 | 50% | 88% |
| *Enchytraeus japonensis* | 0.000 | 0.001 | 0.001 | 0.000 | 0.000 | 0.000 | 33% | 0.000 | 0.000 | 0.000 | 0.000 | 0.000 | 0.000 | 0% | 50% |
| *Drawida sp. Watarase* | 0.000 | 0.000 | 0.000 | 0.000 | 0.000 | 0.000 | 0% | 0.000 | 0.000 | 0.000 | 0.000 | 0.000 | 0.000 | 0% | 25% |
| *Aporrectodea aff.* | 0.011 | 0.000 | 0.000 | 0.036 | 0.010 | 0.009 | 67% | 0.000 | 0.000 | 0.000 | 0.000 | 0.100 | 0.067 | 33% | 75% |
| *Amynthas gracilis* | 0.000 | 0.001 | 0.000 | 0.000 | 0.000 | 0.000 | 17% | 0.000 | 0.000 | 0.000 | 0.000 | 0.000 | 0.000 | 0% | 25% |
| *Drawida koreana* | 0.000 | 0.000 | 0.000 | 0.040 | 0.002 | 0.003 | 50% | 0.000 | 0.000 | 0.000 | 0.000 | 0.000 | 0.000 | 0% | 33% |
| *Bimastos palustris* | 0.000 | 0.000 | 0.000 | 0.000 | 0.000 | 0.000 | 0% | 0.000 | 0.000 | 0.043 | 0.000 | 0.000 | 0.000 | 17% | 29% |
| *Henlea perpusilla* | 0.000 | 0.000 | 0.000 | 0.000 | 0.000 | 0.000 | 0% | 0.000 | 0.000 | 0.000 | 0.000 | 0.000 | 0.000 | 0% | 13% |
| *Amynthas hupeiensis* | 0.000 | 0.000 | 0.000 | 0.000 | 0.002 | 0.004 | 33% | 0.000 | 0.000 | 0.000 | 0.000 | 0.000 | 0.000 | 0% | 21% |
| *Megascolecidae sp.* | 0.000 | 0.000 | 0.000 | 0.000 | 0.000 | 0.000 | 0% | 0.000 | 0.000 | 0.000 | 0.000 | 0.000 | 0.000 | 0% | 13% |
